# Women’s and girls’ experiences of menstruation in low- and middle-income countries: A systematic review and qualitative metasynthesis

**DOI:** 10.1371/journal.pmed.1002803

**Published:** 2019-05-16

**Authors:** Julie Hennegan, Alexandra K. Shannon, Jennifer Rubli, Kellogg J. Schwab, G. J. Melendez-Torres

**Affiliations:** 1 The Water Institute, Department of Environmental Health and Engineering, Johns Hopkins Bloomberg School of Public Health, Baltimore, Maryland, United States of America; 2 Department of Population, Family and Reproductive Health, Johns Hopkins Bloomberg School of Public Health, Baltimore, Maryland, United States of America; 3 Femme International, Majengo, Moshi, Tanzania; 4 Peninsula Technology Assessment Group, College of Medicine and Health, University of Exeter, Exeter, United Kingdom; University of Manchester, UNITED KINGDOM

## Abstract

**Background:**

Attention to women’s and girls’ menstrual needs is critical for global health and gender equality. The importance of this neglected experience has been elucidated by a growing body of qualitative research, which we systematically reviewed and synthesised.

**Methods and findings:**

We undertook systematic searching to identify qualitative studies of women’s and girls’ experiences of menstruation in low- and middle-income countries (LMICs). Of 6,892 citations screened, 76 studies reported in 87 citations were included. Studies captured the experiences of over 6,000 participants from 35 countries. This included 45 studies from sub-Saharan Africa (with the greatest number of studies from Kenya [*n* = 7], Uganda [*n* = 6], and Ethiopia [*n* = 5]), 21 from South Asia (including India [*n* = 12] and Nepal [*n* = 5]), 8 from East Asia and the Pacific, 5 from Latin America and the Caribbean, 5 from the Middle East and North Africa, and 1 study from Europe and Central Asia. Through synthesis, we identified overarching themes and their relationships to develop a directional model of menstrual experience. This model maps distal and proximal antecedents of menstrual experience through to the impacts of this experience on health and well-being. The sociocultural context, including menstrual stigma and gender norms, influenced experiences by limiting knowledge about menstruation, limiting social support, and shaping internalised and externally enforced behavioural expectations. Resource limitations underlay inadequate physical infrastructure to support menstruation, as well as an economic environment restricting access to affordable menstrual materials. Menstrual experience included multiple themes: menstrual practices, perceptions of practices and environments, confidence, shame and distress, and containment of bleeding and odour. These components of experience were interlinked and contributed to negative impacts on women’s and girls’ lives. Impacts included harms to physical and psychological health as well as education and social engagement. Our review is limited by the available studies. Study quality was varied, with 18 studies rated as high, 35 medium, and 23 low trustworthiness. Sampling and analysis tended to be untrustworthy in lower-quality studies. Studies focused on the experiences of adolescent girls were most strongly represented, and we achieved early saturation for this group. Reflecting the focus of menstrual health research globally, there was an absence of studies focused on adult women and those from certain geographical areas.

**Conclusions:**

Through synthesis of extant qualitative studies of menstrual experience, we highlight consistent challenges and developed an integrated model of menstrual experience. This model hypothesises directional pathways that could be tested by future studies and may serve as a framework for program and policy development by highlighting critical antecedents and pathways through which interventions could improve women’s and girls’ health and well-being.

**Review protocol registration:**

The review protocol registration is PROSPERO: CRD42018089581.

## Abstract 2

Julie Hennegan and colleagues reveal the experiences and challenges facing women and girls in low- and middle-income countries when menstruating.

## Author summary

**Why was this study done?:**

- A growing body of qualitative research has highlighted the importance of menstrual experiences for the health and well-being of women and girls in low- and middle-income countries (LMICs).
- Qualitative research has identified an array of factors contributing to experiences but has not developed clear theory to direct intervention and evaluation.

**What did the researchers do and find?:**

- We systematically searched and critically appraised the body of qualitative studies of menstrual experience in LMICs.
- We identified overarching themes and mapped the relationships between them to develop a directional model of menstrual experience.
- Women and girls reported impacts of negative menstrual experiences on physical and psychological health, education, employment, and social participation.
- Both resource limitations and the sociocultural context contribute to menstrual experience.

**What do these findings mean?:**

- Women’s and girls’ menstrual experiences are complex and multifaceted, but across LMICs, consistent factors contribute to experiences.
- Findings advance the development of problem theory in menstrual health, and the developed model can be used as a framework for developing interventions and evaluation.
- Future interventions should seek to address identified antecedents of menstrual experience, including knowledge, social support, restrictive behavioural expectations, and the physical and economic environment.

## Introduction

Each day, more than 300 million women are menstruating [1]. There is increasing recognition that this natural process is experienced negatively and presents a barrier to health and gender equality in low- and middle-income contexts [2]. A growing body of qualitative research has been critical to highlighting this issue. Early studies focused on adolescent girls reported that menstruation was experienced with discomfort and fear [3–5]. Access to clean, reliable materials to absorb menses, supportive sanitation infrastructure, and biological and pragmatic information about menstruation were highlighted as core challenges [6, 7]. Studies suggested that these challenges negatively impacted school participation [4, 8, 9], health, and well-being [10, 11, 12, 13]. Fewer studies of adult women have highlighted that they too lack resources and support [14, 15], which may contribute to stress and absence from employment [16, 17].

In response to growing advocacy, programs and policies seeking to address menstrual needs have emerged rapidly. Development of these interventions has drawn largely on qualitative literature, with limited high-quality quantitative research and controlled trials available [18, 19]. However, this use of qualitative evidence has been anecdotal and situated, drawing on individual studies that may be restricted in scope and have limited generalizability beyond their context. Thus, the first objective of this review was to undertake a systematic search and synthesis of extant qualitative studies to draw out common themes, as well as appraise the coverage and quality of existing research.

The second objective of this review was to draw on the rich body of qualitative evidence to advance understanding of menstrual health. Program-orientated scoping research has often identified lists of factors important for menstrual experience, alongside lists of consequences for health and education. However, pathways between the variety of contributors and their impacts remain poorly understood [20]. Menstrual health research has tended to draw on programmatic models rather than detailed problem theory. One common model includes a Venn diagram with three circles: knowledge, menstrual products, and sanitation (e.g., [21, 22]). This may also include cultural norms and social factors as a broad concept surrounding the three core circles. These Venn models provide an intuitive high-level picture and pillars to inform programming. However, they do not provide a detailed problem theory, mapping relationships and aetiological pathways to hypothesised impacts on health and education. For example, we would expect different causal pathways contributing to reproductive tract infections than to mental health. The need for detailed problem theory becomes more salient when, in responding to the many challenges for women and girls, actors develop increasingly complex, multicomponent interventions, with many desired outcomes.

A lack of clarity around core concepts, or terminologies, in menstrual research has further complicated the development of problem theory. Early efforts were united around ‘menstrual hygiene’. This was defined as ‘women and adolescent girls using a clean menstrual management material to absorb or collect blood that can be changed in privacy as often as necessary for the duration of the menstruation period, using soap and water for washing the body as required, and having access to facilities to dispose of used menstrual management materials’ [6], providing a target for improving the effective and hygienic management of menses. However, because this definition does not include other menstrual needs highlighted by qualitative studies, more recent versions have expanded the definition, adding that ‘[women and adolescent girls] understand the basic facts linked to the menstrual cycle and how to manage it with dignity and without discomfort or fear’ [23]. Dissatisfied with the coverage and physical focus of ‘menstrual hygiene’, more recent efforts have discussed ‘menstrual health’, suggested as ‘an encompassing term that includes both menstrual hygiene management (MHM) as well as the broader systemic factors that link menstruation with health, well-being, gender, education, equity, empowerment, and rights’ [21]. This term may be useful to describe a broad field of research and practice and facilitates the consideration of menstrual disorders (e.g., dysmenorrhea, endometriosis). However, although the use of ‘menstrual health’ or expansions to ‘menstrual hygiene’ may be beneficial for more comprehensive advocacy, neither addresses the underlying lack of unified problem theory. Thus, through an interpretive approach in this review, we seek to evolve problem theory and concept definitions by providing a more nuanced model and iteratively identifying aspects of menstrual experience.

In sum, this systematic review and metasynthesis had 2 overarching objectives: (1) synthesise and appraise the extant qualitative research on women’s and girls’ menstrual experiences and (2) interpret findings across studies to develop an integrated model of menstrual experience mapping relationships between contributing factors (antecedents), menstrual experience, and the consequences of poor experiences for health and well-being (impacts).

## Methods

The review protocol is registered on PROSPERO: CRD42018089581 and is reported according to PRISMA guidance (S1 PRISMA Checklist).

### Search strategy and selection criteria

The search strategy was designed to capture studies reporting on women’s and girls’ experiences of menstruation. Searches were undertaken in 11 databases (Applied Social Science Index and Abstracts, Cumulative Index of Nursing and Allied Health Literature, ProQuest Dissertation and Theses, Embase, Global Health, Medline, Open Grey, Popline, PsycINFO, Sociological Abstracts, WHO Global Health Library) using a prespecified, piloted strategy reported in Table 1. Searches were completed in January 2019 with no language of publication or date restrictions applied. Comprehensive grey literature searching and hand searching were undertaken. Organisations attending to menstrual health were identified through participation in reports [7, 21], stakeholder meetings [2], and online searches. Websites (see list in S1 Text) were searched using relevant terms (e.g., ‘menstrual’, ‘menstruation’). Citations of included studies and reference lists of large menstrual health reports were searched [7, 21]. Results were exported into EPPI-Reviewer 4 (EPPI-Centre; https://eppi.ioe.ac.uk/cms/Default.aspx?tabid=2914). Two authors (JH, AS) independently screened titles and abstracts, followed by full-text screening to determine eligibility (JH).

**Table 1:** Embase search strategy.

**Search 1**: **Menstrual**	‘menstruation’/exp OR ‘menarche’/exp OR (‘menstrual period’ OR menstru* OR menses OR catamenia OR menarche):ti,ab,kw
**Search 2**: **Experience**	‘social behavior’/exp OR ‘experience’/exp OR ‘comprehension’/exp OR (knowledge OR comprehen* OR attitude* OR practice* OR experienc* OR perception* OR understand* OR challenge* OR barrier OR facilitat* OR impact OR affect OR effect):ti,ab,kw
**Search 3**: **Qualitative research methods**	‘qualitative research’/exp OR ‘interview’/exp OR (qualitative* OR ‘focus group’ OR ‘focus groups’ OR focus-group* OR interview* OR ‘semi-structured interview’ OR ‘semi-structured interviews’ OR ‘unstructured interview’ OR ‘unstructured interviews’ OR ‘thematic analys*’ OR ethnograph* OR ‘grounded theory’ OR ‘narrative’ OR ‘interpretive’ OR ‘discourse analys*’ OR ‘content analys*’ OR ‘framework analys*’ OR interpetiv* OR interpretativ* OR phenomeno* OR ‘mixed-method’ OR ‘mixed method’ OR ‘mixed-methods’ OR ‘mixed methods’):ti,ab,kw
**Final search**	1 AND 2 AND 3

Studies were eligible if they reported qualitative analysis of the menstrual experiences of women and girls residing in low- or middle-income countries (LMICs) as defined by the World Bank [24]. Studies that included women from LMICs now residing in high-income countries, or that combined populations from LMICs with those in high-income settings, were excluded. While these experiences also deserve increased attention, this review sought to synthesise the large set of studies situated in LMICs to inform evolving policy and practice in these regions. Studies exclusively concerning the acceptability of menstrual suppression were excluded. Studies focussed on puberty more broadly, or the use of sanitation infrastructure, were only included when they reported on experiences of menstruation. For example, studies that included lists of puberty education needs that referenced menstruation but did not report on women’s or girls’ lived menstrual experiences were not included. Similarly, studies focussed on menopause, premenstrual syndrome, or polycystic ovary syndrome were not eligible for inclusion. Studies capturing the menstrual experiences of populations with menstrual disorders (e.g., dysmenorrhea, endometriosis) were eligible. Menstruating women and girls were the target population; thus, studies were excluded if they focused exclusively on girls’ premenarche, key informants, or males. Where key informant interviews were analysed alongside women’s and girls’ experiences, studies were included, but analysis focused on the experience of the target population. Qualitative and mixed-methods studies reported in peer-reviewed or grey literature were eligible for inclusion. Studies were excluded if they did not report any qualitative analysis or results (e.g., qualitative responses were back-coded for quantitative description).

Following full-text screening, study research questions were extracted and iteratively grouped. Three groupings emerged: studies broadly focused on menstrual experiences, studies of experiences of menstruation for those with dysmenorrhea or disorders, and studies of experiences of menstrual interventions or products. Because the review aimed to provide a synthesis of menstrual experience and advance problem theory rather than explore the role of interventions, the third grouping was excluded from the present review but was retained for analyses reported elsewhere.

### Study quality appraisal

Included studies were appraised using the EPPI-Centre checklist [25]. This checklist assesses methodological quality across recommended domains: sampling, data collection, analysis, interpretation, and privileging of participant perspectives (see S1 Table) [26, 27]. The checklist also facilitates generation of overall trustworthiness taking into account the first 4 ratings (sampling, data collection, analysis, and support for interpretation), as well as relevance to the review (relevance to the review question, privileging and involvement of participant perspectives), rated as low, medium or high. One author (JH) appraised the quality of all studies; 10% were independently appraised by a second author (GJMT), with 100% agreement.

### Analysis

Reflecting the research questions, we employed a thematic synthesis approach [28], or an approach that seeks to describe themes as recurrent ‘units of meaning’ within and across studies, to synthesise insights from individual studies [29, 30]. We then used these themes to generate thematic networks to facilitate repeated comparison of the relationships between these themes. The use of thematic networks was appropriate as it includes (a) the explicit step of evidencing themes against specific statements and quotes from included studies and (b) the development of relationships between themes to form a network. The use of thematic networks reflected a lines-of-argument approach to meta-ethnography [31]; that is, drawing on the synthesis of qualitative research (meta-ethnography), we sought to go beyond merely describing a phenomenon to interpreting and understanding the social processes underlying it as part of an integrated scheme, such that no one study captures the entirety of the phenomenon (lines of argument).

Analysis was undertaken in 4 steps:

1. The findings of high- and medium-quality studies were thematically coded using NVivo 12 (QSR International; https://www.qsrinternational.com/nvivo/nvivo-products) by the first author. Throughout initial coding, multiple mappings of the relationships between themes were generated. Representative quotations and core sections of text corresponding to each theme were recorded for each included study.
2. Emergent themes were discussed among the authorship team, and multiple mappings of the relationships between themes were generated until authors reached consensus on a representation with the greatest explanatory power.
3. Findings of low-quality studies were assessed for their fit with the proposed themes and integrated model. Low-quality studies supported the primary analysis, and no new constructs emerged. This is akin to the ‘sensitivity analysis’ common in systematic reviews of qualitative research, in which themes are checked to see if they rely on low-quality studies alone [30].
4. Using Nvivo 12, all studies were recoded against the consensus themes and integrated model for validation.

## Results

**Fig 1:**
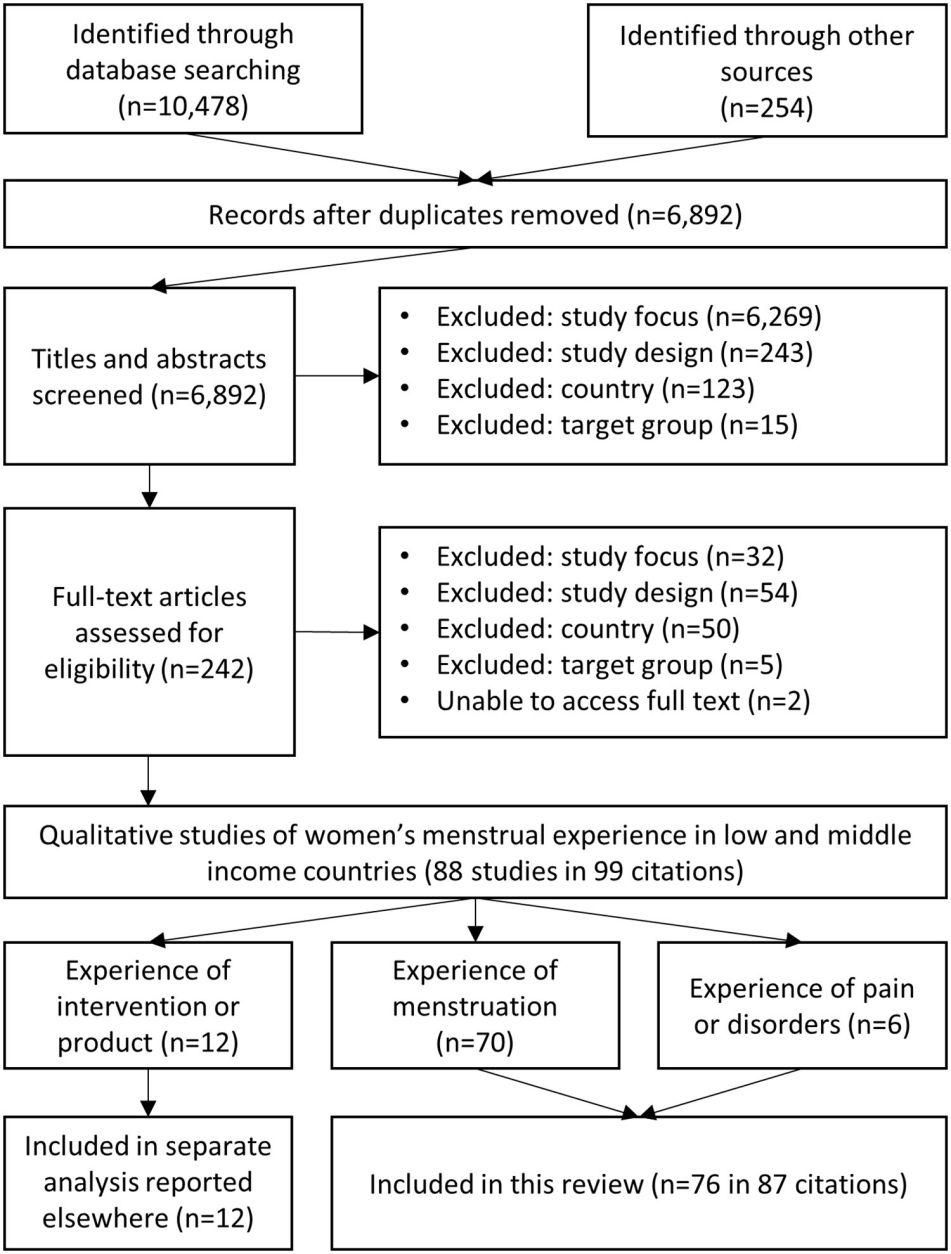
Review flow diagram showing the number of titles, abstracts, and full-text citations assessed for eligibility and reasons for exclusion.

The review flowchart is presented in Fig 1.

Study characteristics, quality, and relevance are presented in Table 2. Included studies captured experiences from 35 countries and over 6,000 participants. India was the country with the highest number of studies (*n* = 12), followed by Kenya (*n* = 7) and Uganda (*n* = 6).

Using World Bank regional groupings [24], most studies were conducted in sub-Saharan Africa (*n* = 45); this was followed by 21 studies undertaken in South Asia, 8 in East Asia and the Pacific, 5 in Latin America and the Caribbean, 5 in the Middle East and North Africa, and 1 study from Europe and Central Asia (Kyrgyzstan).

**Table 2:** Included study characteristics.

Study ID	Study year	Country (area)	Population	No. participants (individual; FGD)^1^	Age	Data collection method	Analytic approach	Trustworthiness	Relevance
**Adegbayi 2017** [32]	NR	Nigeria (Ede)	University students	136	16–25	Written narrative	Narrative	Medium	Medium
**Al Omari 2016** [33]	2014	Jordan (Az-Zarqua)	Girls in school	7	12–14	Journal written daily for 1 week	Thematic	Medium	High
**Al-Sabir 1998** [34]	1997	Bangladesh (Bogra)	Women and girls	108	10–45	IDI; FGD	NR	Medium	Medium
**Al-Shurbji 2017** [35]	NR	Jordan (Za’atari)	Women	17	19–40	IDI	Grounded theory	Low	Medium
**Amatya 2018** [36]	NR	Nepal (Achham)	Girls in school	1 FGD (*n* = 7)	13–19	FGD	Phenomenological	Low	Medium
**Behera 2015** [37]	2012	India (Maharashtra)	Girls in school	32	14–15	FGD	Thematic	Medium	Medium
**Bilandi 2015** [38]	2013	Iran (South Khorasan)	Women	14	18–45	IDI	Grounded theory	Medium	Medium
**Boosey 2014**[39]	2013	Uganda (Rukungiri)	Girls in school	6 FGDs (6–9 participants)	13–16	Mixed methods; FGD	Thematic	Low	Low
**Budhathoki 2018** [40]	2015	Nepal (Sindhupalchowk)	Women and girls in temporary shelters	5	15–49	Mixed methods; IDI	Thematic	High	Medium
**Caruso 2013** [41]	2012	Sierra Leone (Freetown)	School-aged girls^2^	20; 8 FGDs (*n* = NR)	School age	IDI; FGD	NR; themes reported	Medium	High
**Caruso 2017** [16]	2014	India (Odisha)	Women	8 FGDs (*n* = 46)	18–70	Free list interview; FGD	Thematic	High	Medium
**Castaneda 1996** [42]	NR	Mexico (Morelos)	Women	NR	NR	FGD; IDI; observation	Ethnography	Medium	Low
**Chebii 2018** [43]	NR	Kenya (Nairobi county)	Girls in school	18 (in FGDs and IDIs)	15–17	FGD, IDI	NR; grounded theory	High	High
**Chinyama 2019** [44, 45]	2015	Zambia (Mumbwa, Rufunsa)	Girls in school	12; 6 FGDs (*n* = 48)	14–18	FGD, IDI	Thematic	High	High
**Chothe 2014** [46]	NR	India (Pune)	Girls in school	381	9–13	Students posed questions to doctor	NR	Medium	Low
**Crawford 2014** [47]	NR	Nepal (Kathmandu)	Women	2 FGDs (*n* = 8)	20–50	FGD; IDI	NR; themes reported	Medium	Medium
**Crichton 2013** [10, 48–50]	2008	Kenya (Nairobi)	Women and girls	29; 18 FGDs (*n* = NR)	12–17, adult	IDI; FGD	Thematic	High	High
**Daniels 2016** [51]	NR	Cambodia (Banteay Meanchey; Kratie)	Women and girls	165; 24 FGDs (*n* = 180)	14+	Mixed methods; IDI; FGD; observation	Grounded theory	High	High
**da Silva Bretas 2011** [52]	NR	Brazil (Embu das Artes)	Girls in school	1 FGD (repeated ×3) (*n* = 17)	14–18	FGD	Categorical content (thematic)	Medium	Low
**Devnarain 2011** [53]	2009	South Africa (KwaZulu-Natal)	Girls in school	NR	NR	FGD; participatory activities (daily clock)	Thematic	Medium	Low
**Dhingra 2009** [54]	NR	India (Jammu)	School-aged girls	131	13–15	IDI; small group	Content analysis	Low	Low
**Do Amaral 2011** [55]	NR	Brazil (Campinas)	Women	8 FGDs (*n* = 64)	21–51	FGD	Thematic	Medium	Medium
**Dolan 2014** [4]	2008	Ghana	School-aged girls	99; FGDs (*n* = 136)	NR	IDI; FGD	NR	Low	Medium
**Ellis 2016** [56, 57]	2013	Philippines (Masbate; Manila)	Girls in schools	FGDs (*n* = 79)	11–18	FGD	NR; themes reported	Medium	High
**Garg 2001** [14]	1996–2000	India (Delhi)	Women	52	19–44	IDI	NR; themes reported	Medium	High
**Girod 2017** [58]	2015	Kenya (Nairobi)	Girls in school	6 FGDs (*n* = NR)	Grade 6–8	FGD; participatory activities (ideal place to manage period; imagining life of a normal girl)	Thematic	High	High
**Guerry 2013** [59]	2013	Uganda (Kisinga; Bwera)	Girls in school	7	12–14	Mixed methods; IDI	Thematic	Medium	Medium
**Hosseini 2018** [60]	NR	Iran (Tehran)	University students	8	21–25	IDI	Interpretive phenomenological	Medium	Medium
**Ismail 2016** [61]	NR	South Africa (Western Cape)	University students	16	18–23	FGD	Discourse	Low	Medium
**IWDA 2017** [62, 63]	2017	Solomon Islands; Fiji; Papua New Guinea	Women and girls	8; 25 FGDs (*n* = 233)	13–70	FGD; IDI; observation; participatory activities (10-seed, ideal latrine)	Thematic	Medium	Medium
**Jewitt 2014** [64]	NR	Kenya (Kisumu)	School-aged girls	7; 7 FGDs (*n* = 53)	13–25	IDI; FGD; participatory (ranking difficulties; ideal toilet)	NR	Medium	Hedium
**Kansal 2016** [65]	2011	India (Varanasi)	School-aged girls	4 FGDs (*n* = 40)	15–19	FGD	NR; thematic	Low	Low
**Krishnan 2016** [66]	2013	India (Assam; Odisha)	Women	16; 7 FGDs (*n* = NR)	19–35	FGD; IDI; participatory (transect walks, priority ranking)	Thematic	Low	Low
**Kyomugisha 1999** [67]	NR	Uganda	School-aged girls	75	NR	FGD; IDI	NR	Low	Low
**Lahme 2016** [68, 69]	2013	Zambia (Mongu)	Girls in school	6 FGDs (*n* = 51)	13–20	FGD	Thematic	High	High
**Long 2013** [9]	2012	Bolivia (CoChabamba)	Girls in school	11; 12 FGDs (*n* = 60)	Secondary school	FGD; IDI	NR; framework reported	Medium	High
**Mason 2013** [11]	NR	Kenya (Nyanza)	Girls in school	11 FGDs (*n* = 120)	14–16	FGD	Thematic	High	High
**McMahon 2011** [5]	NR	Kenya (Nyanza)	Girls in school	48	12–16	IDI; FGD; observation	NR; themes reported	Medium	High
**Miiro 2018** [70]	2016	Uganda (Entebbe)	Girls in school	16; 8 FGDs (*n* = NR)	15–16	IDI; FGD; participatory (NR)	Thematic	Medium	High
**Morowatisharifabad 2018** [71]	2016–2017	Iran (Bam city)	Women and girls in school	26	13–15; 31–48	IDI	Directed content	Medium	Medium
**Morrison 2016** [72]	NR	Nepal (Udaypur; Sindhuli)	Girls in school	32; 4 FGDs (*n* = 25)	Mean = 15	FGD; pair interview	NR; themes reported	High	High
**Mumtaz 2016** [73]	2015	Pakistan (Baluchistan)	School-aged girls	177	16–19	FGD; participatory activities (menstrual stories, ‘perfect toilet’ and suggested sanitation improvements, design puberty curriculum)	Latent content analysis	Medium	High
**Naeem 2015** [74]	2014	Pakistan	Girls in school	FGDs (*n* = 124)	Grade 6–9	FGD; observation	NR	Low	Low
**Nanda 2016** [75]	2015	Zambia (Eastern Province)	Girls in school	16 FGDs (*n* = NR)	12–15	FGD	NR; themes reported	Medium	High
**Narayan 2001** [76]	2001	India (Pondicherry)	Girls in school, Women	60 IDIs; 1 FGD (*n* = NR)	12–17; NR	IDI; FGD; participatory activities (body mapping)	NR	Low	Low
**Nechitilo 2016** [77]	2015	Kyrgyzstan (Chyu, Osh)	Girls in School, Women	6 IDIs; 8 FGDs (*n* = 48)	NR	FGD; IDI; observation	NR; deductive themes	Medium	High
**Padmanadbhanunni 2017** [78]	NR	South Africa (Eastern Cape; Western Cape)	University students	5; 3 FGDs (*n* = 15)	20–27	FGD; IDI	Thematic	Medium	Medium
**Parker 2014** [15]	2006	Uganda (Katakwi)	Women and girls (internally displaced)	29 FGDs (*n* = 85)	9–20	FGD; IDI	NR; stated critical realist perspective	Medium	High
**Pillitteri 2011** [79]	2011	Malawi	Girls in school	7 FGDs (*n* = NR)	14–21	FGD	NR; themes reported	Medium	Medium
**Person 2014** [80]	NR	Zambia (Choma; Kasama; Chongwe)	Girls in school	24 IDIs; FGDs (*n* = 101)	12–18	FGD; IDI	NR; themes reported	Low	Medium
**Rheinlander 2018** [81]	2014	Ghana (Ningo-Prampram)	Girls in school	4 FGDs (*n* = 33)	15–23	FGD	Thematic	High	High
**Sanduvac 2017** [82]	NR	Tanzania (Nduta Camp)	School-aged girls	1 FGD (*n* = 15)	NR	Mixed methods; FGD	NR	Low	Low
**Schmitt 2017** [83]	2015	Myanmar; Lebanon	Women and girls (refugee)	149 (in FGDs)	14–49	FGD; participatory mapping	Thematic	High	High
**Scorgie 2016** [84]	2012	South Africa (Durban)	Women	7; 1 FGD (*n* = 12)	18–35	IDI; participatory activity (photovoice)	Grounded theory	High	High
**Secor-Turner 2016** [85]	NR	Kenya (Tharaka-Nithi)	Girls in school	29	13–21	IDI	Descriptive content analysis	Medium	Medium
**Singh 2006** [86]	NR	India (Rural North)	Women	4 FGDs (4–6 participants)	20–40	Mixed methods; FGD	NR; themes reported	Low	Medium
**Sommer 2009** [3, 8, 87]	2007	Tanzania (Kilimanjaro)	School-aged girls	16; 4 FGDs (repeated) (*n* = 80)	16–19	IDI; FGD; participatory activities (menstrual narratives, puberty questions, design puberty curriculum, recommended improvements to school environment, draw ‘perfect’ toilet)	Grounded theory	High	High
**Sommer 2015** [88, 89]	2012	Tanzania (Kilamanjaro); Ghana (Accra); Cambodia (Phnom Penh; Battambang); Ethiopia (Oromia; Amhara)	School-aged girls	4 FGDs (repeated) per country (*n* = 150, *N* = 600)	16–19	IDI; FGD; participatory activities (menstrual stories, brainstorm school environment improvements, draw ‘perfect’ toilet)	Grounded theory	High	High
**Sosa-Sanchez 2014** [90]	2009	Mexico (Morelos)	Women	22	18–52	IDI	Grounded theory	High	High
**Timiru 2015**[91, 92]	2015	Ethiopia; South Sudan; Uganda; Tanzania; Zimbabwe	Girls in school	Unclear, >480 IDIs; >12 FGDs (*n* > 500)	11+	Mixed methods; IDI; FGD	Thematic	Low	Low
**Tegegne 2014** [93]	2013	Ethiopia (North East)	Girls in school	9; 4 FGDs (*n* = NR)	12–19	Mixed methods; IDI; FGD	Inductive content analysis	Medium	Medium
**Thakur 2014** [94]	2008	India (Mumbai)	Women and girls	5 FGDs (*n* = NR)	School-aged to 29	FGD	Content	Low	Low
**Trinies 2015** [95]	NR	Mali (Sikasso; Koulikoro)	Girls in school	26	12–17	IDI	Thematic	Medium	Medium
**Ullrich 1992** [96]	1964–1985	India (Karnataka)	Women	NR	NR	IDI; observation	Ethnography	Low	Low
**Umeora 2008** [97]	2005	Nigeria (Igbo)	Women	12	50+	Mixed methods; IDI	NR	Low	Low
**UN Women 2017** [98]	NR	Niger (Tahoua; Maradi; Tillaberi; Zinder)	Women and girls	10 FGDs (*n* = NR)	15–49	Mixed methods; IDI; FGD	NR	Low	Medium
**Wall 2016** [99]	2015	Ethiopia (Tigray)	Women	349	10+	Mixed methods; IDI	NR; themes reported	Low	Low
**Wall 2018** [100]	2015	Ethiopia (Tigray)	Women and girls	16 IDIs; FGDs (*n* = 96)	NR	FGD; IDI	NR; thematic	Medium	Medium
**WaterAid 2009** [101]	2009	Nepal (Dhading, Morang, Lalitpur; Kathmandu)	Girls in school	5; 4 FGDs (*n* = 47)	12–20	Mixed methods; FGD; IDI	Thematic	Medium	Medium
**WSSCC 2014** [102]	2014	Senegal (Louga)	Women and girls	359	13–65	FGD; IDI	NR	Low	Medium
***Disorders and pain***									
**Aziato 2014** [103, 104]	NR	Ghana (Accra)	University students; Girls in school	16	16–38	IDI	Content analysis	High	Medium
**Hemachandra 2009** [105]	2007	Sri-Lanka (Ratnapura)	Women	5; 6 FGDs (*n* = 48)	15–49	FGD; IDI	Phenomenological	High	Medium
**Kavitha 2014** [106]	NR	India (Tamil Nandu)	School-aged girls	10	9th standard	IDI	Phenomenological	Low	Low
**Titilayo 2009** [107]	2007	Nigeria (Ile-Ife)	University students	37	20–24	Mixed methods; IDI	Grounded theory	Medium	Medium
**Walraven 2002** [108]	1999	The Gambia (Farafenni)	Women	10	15–54	IDI	NR	Low	Low
**Wong 2011** [109]	2009	Malaysia (Kuala Lumpur; Kelantan)	Girls in school	27 FGDs (*n* = 172)	13–19	FGD	Thematic	Medium	Medium

^1^Indicates participants captured individually, followed by total number of FGDs and number of participants included in FGDs or per FGD as reported in included studies. *N*’s reported reflect only the population of interest (women and girls) and do not include data collected from key informants or males.

^2^Indicates girls of school-going age were sampled, including both those attending and not attending school.

**Abbreviations**: FGD, Focus Group Discussion; IDI, individual (in-depth) interview; NR, not reported.

Focus Group Discussions (FGDs) were the most common data collection method used, particularly when working with adolescent girls. Eleven studies reported using participatory activities such as asking groups to design an ‘ideal latrine’, or rank priorities for improvements to explore experiences and preferences. A total of 55 studies included adolescent girls (school aged), with only 21 studies including exclusively university students or adult women. Most studies reported undertaking a thematic approach to analysis.

Overall study quality and relevance ratings are reported in Table 2. Classifications according to each item in the EPPI-Centre checklist, along with justifications, are displayed in S1 Table. Most studies were situated within a public health discipline and focused on cataloguing antecedents and impacts of menstruation on participants’ lives, rather than sociological analysis. Study quality was varied, with 18 studies rated as high, 35 as medium, and 23 as low trustworthiness. Lower-quality studies were characterised by poor reporting of participant selection and analysis, with many having no defined or described analytic approach. After initial analysis of high- and medium-quality studies, analysis and integration of lower-quality studies did not elicit any new themes nor changes to the integrated model.

### Developing an integrated model of menstrual experience

**Fig 2:**
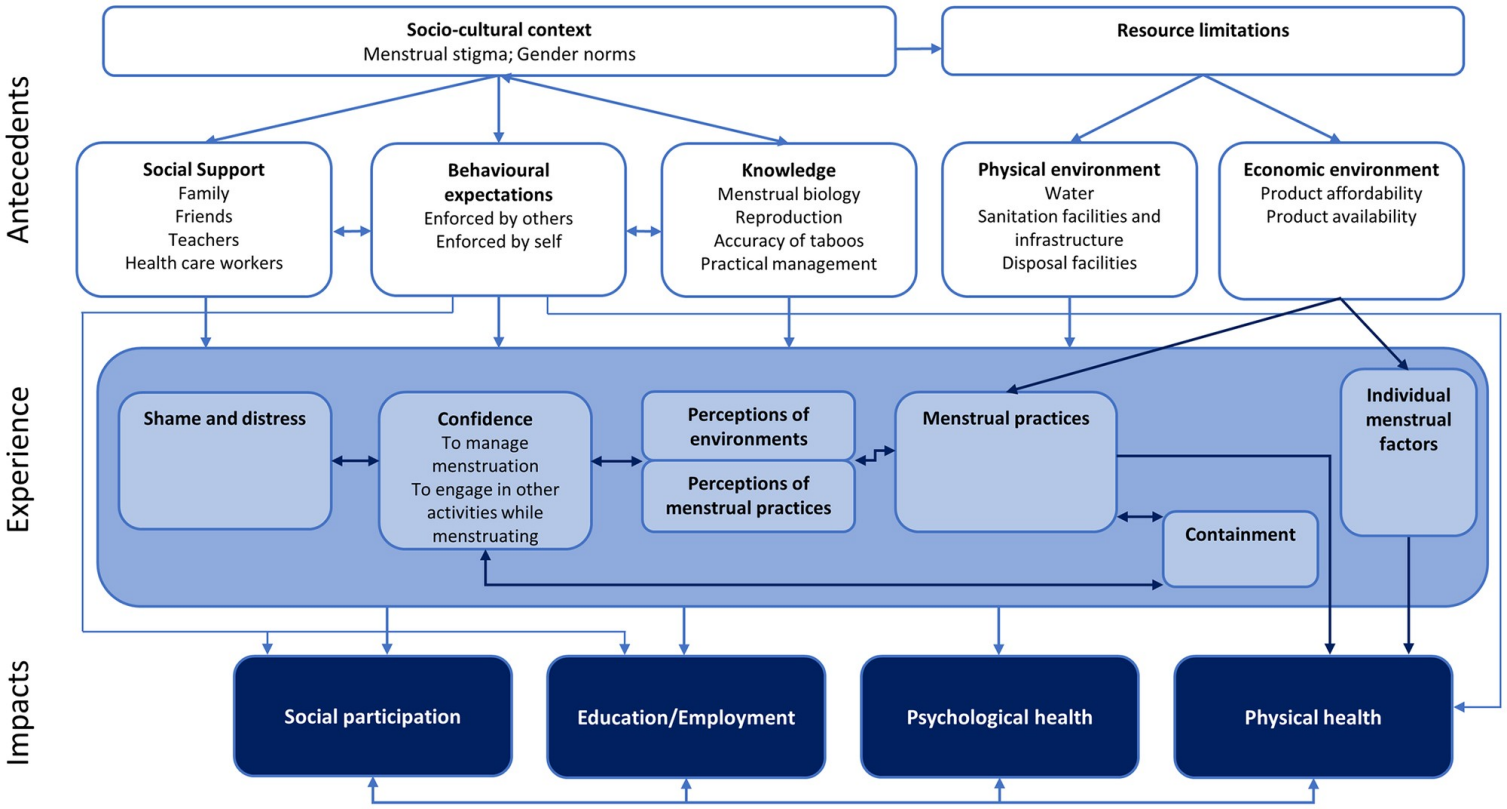
Integrated model of menstrual experience. Bolded headings capture themes, with subthemes presented below in unbolded text. Arrows depict directional and bidirectional relationships between themes.

Fig 2 presents an integrated model of menstrual experience. The figure summarises the themes and subthemes identified through analysis and the relationships between them. Studies varied in their coverage of included themes, with none reflecting the full picture gained through metasynthesis.

### Author orientations

In identifying themes and mapping the relationships between themes as part of our analysis, differences in author orientations across the body of literature were apparent. We found that studies varied along a continuum in the extent to which they approached menstrual experiences as a result of the sociocultural context, particularly menstrual stigma, or focused on resource deficits in the environment. These orientations influenced the focus of studies, topics covered, and author interpretation of the relationships between antecedents, experience, and impacts.

Studies with a more stigma-centric orientation conceptualised menstrual experiences and negative outcomes as resulting from the stigmatised and gendered nature of menstruation. These studies were often those using individual interviews or engaging adult women to provide reflexive insights into their experiences. Themes centred around the pressure to suppress evidence of menses and control the body. Studies focused on stigma, knowledge, and social support as antecedents of experience. Where resource limitations were discussed in these studies, they were considered problematic because they failed to support socially proscribed discretion. Menstrual experiences were characterised by shame and a lack of confidence to engage socially when menstruating. Stigma-centric models of menstruation rarely elaborated on physical health consequences; rather, they detailed psychological and educational concerns and consequences for empowerment. Others did not address the impacts of menstruation on such outcomes, exploring only the experience itself.

Other studies were orientated towards poverty and resource limitations as causes of menstrual experiences and impacts. These studies viewed the resources available to women and girls as inadequate to meet their basic biological needs. This included an emphasis on menstrual hygiene practices such as the use of materials poorly suited to absorbing menses. Knowledge and cultural practices were important in resource-centric studies but were more often linked with a failure to support effective management practices. Menstrual hygiene, the hygienic and effective management of menstrual blood, lay at the centre of experience in these studies, which frequently noted concerns around infection and consequences for school attendance.

Our final integrated model synthesised studies along this continuum. As displayed in Fig 2, the model views resource limitations and the sociocultural context, including menstrual stigma, as determinants of menstrual experience and negative impacts. Themes are described from this perspective.

Table 3 summarises the individual studies contributing to each theme according to their level of trustworthiness.

**Table 3:** Summary table of studies contributing to each theme according to level of trustworthiness.

Theme	High trustworthiness	Medium trustworthiness	Low trustworthiness
**ANTECEDENTS**			
**Sociocultural Context**			
***Sociocultural context*: *Menstrual stigma***	(*n* = 9) Chebii 2018 [43]; Lahme 2016 [68, 69]; Mason 2013 [11]; Morrison 2016 [72]; Rheinlander 2018 [81]; Scorgie 2016 [84]; Sommer 2009 [3, 8, 87]; Sommer 2015 [88, 89]; Sosa-Sanchez [90]	(*n* = 18) Adegbayi 2017 [32]; Al Omari 2016 [33]; Bilandi 2015 [38]; Caruso 2013 [41]; da Silva Bretas [52]; Do Amaral 2011 [55]; Garg 2001 [14]; Hosseini 2018 [60]; IWDA 2017 [62, 63]; Jewitt 2014 [64]; Long 2013 [9]; McMahon 2011 [5]; Miiro 2018 [70]; Nechitilo 2016 [77]; Padmanabhanunni 2017 [78]; Secor-Turner 2016 [85]; Wall 2018 [100]; WaterAid 2009 [101]	(*n* = 10) Boosey 2014 [39]; Dolan 2014 [4]; Krishnan 2016 [66]; Kyomugisha 1999 [67]; Naeem 2015 [74]; Person 2014 [80]; Tamiru 2015 [91]; Ullrich 1992 [96]; UN Women 2017 [98]; WSSCC 2014 [102]
***Sociocultural context*: *Gender norms***	(*n* = 7) Caruso 2017 [16]; Girod 2017 [58]; Lahme 2016 [68, 69]; Mason 2013 [11]; Rheinlander 2018 [81]; Sommer 2009 [3, 8, 87]; Sosa-Sanchez [90]	(*n* = 8) Al-Sabir 1998 [34]; Castaneda 1996 [42]; Do Amaral 2011 [55]; Ellis 2016 [57]; Hosseini 2018 [60]; Jewitt 2014 [64]; Padmanabhanunni 2017 [78]; Secor-Turner 2016 [85]	(*n* = 2) Dolan 2014 [4]; Ullrich 1992 [96]
***Social support***	(*n* = 14) Aziato 2014 [103, 104]; Chebii 2018 [43]; Chinyama 2019 [44]; Crichton 2013 [10]; Daniels 2016 [51]; Girod 2017 [58]; Hemachandra 2009 [105]; Lahme 2016 [68, 69]; Mason 2013 [11]; Morrison 2018 [72]; Rheinlander 2018 [81]; Scorgie 2016 [84]; Sommer 2009 [3, 8, 87]; Sommer 2015 [88, 89]	(*n* = 27) Adegbayi 2017 [32]; Al Omari 2016 [33]; Al-Sabir 1998 [34]; Behera 2015 [37]; Caruso 2013 [41]; Castaneda 1996 [42]; Crawford 2014 [47]; Do Amaral 2011 [55]; Devnarain 2011 [53]; Ellis 2016 [56, 57]; Garg 2001 [14]; Hosseini 2018 [60]; IWDA 2017 [62, 63]; Jewitt 2014 [64]; Long 2013 [9]; McMahon 2011 [5]; Miiro 2018 [70]; Morowatisharifabad 2018 [71]; Mumtaz 2016 [73]; Nanda 2016 [75]; Nechitilo 2016 [77]; Parker 2014 [15]; Pillitteri 2011 [79]; Tegegne 2014 [93]; Trinies 2015 [95]; WaterAid 2009 [101]; Wong 2011 [109]	(*n* = 9) Boosey 2014 [39]; Dolan 2014 [4]; Kansal 2016 [65]; Kyomugisha 1999 [67]; Naeem 2015 [74]; Person 2014 [80]; Sanduvac 2017 [82]; Singh 2006 [86]; WSSCC 2014 [102]
***Behavioural expectations*: *Externally enforced***	(*n* = 12) Caruso 2017 [16]; Chebii 2018 [43]; Chinyama 2019 [44]; Crichton 2013 [10]; Daniels 2016 [51]; Girod 2017 [58]; Lahme 2016 [68, 69]; Morrison 2018 [72]; Rheinlander 2018 [81]; Sommer 2009 [3, 8, 87]; Sommer 2015 [88, 89]; Sosa-Sanchez [90]	(*n* = 20) Adegbayi 2017 [32]; Al Omari 2016 [33]; Al-Sabir 1998 [34]; Behera 2015 [37]; Caruso 2013 [41]; Crawford 2014 [47]; da Silva Bretas 2011 [52]; Do Amaral 2011 [55]; Ellis 2016 [56, 57]; Garg 2001 [14]; Hosseini 2018 [60]; IWDA 2017 [62, 63]; Jewitt 2014 [64]; Long 2013 [9]; Morowatisharifabad 2018 [71]; Mumtaz 2016 [73]; Nechitilo 2016 [77]; Padmanabhanunni 2017 [78]; Wall 2018 [100]; WaterAid 2009 [101]	(*n* = 14) Al-Shurbji 2017 [35]; Amatya 2018 [36]; Dhingra 2009 [54]; Ismail 2016 [61]; Krishnan 2016 [66]; Narayan 2001 [76]; Person 2014 [80]; Singh 2006 [86]; Tamiru 2015 [91]; Ullrich 1992 [96]; Umeora 2008 [97]; UN Women 2017 [98]; Wall 2016 [99]; WSSCC 2014 [102]
***Behavioural expectations*: *Internally enforced***	(*n* = 8) Caruso 2017 [16]; Chebii 2018 [43]; Daniels 2016 [51]; Hemachandra 2009 [105]; Rheinlander 2018 [81]; Scorgie 2016 [84]; Sommer 2015 [88, 89]; Sosa-Sanchez [90]	(*n* = 10) Caruso 2013 [41]; Castaneda 1996 [42]; Crawford 2014 [47]; da Silva Bretas 2011 [52]; IWDA [62, 63]; Jewitt 2014 [64]; Nechitilo 2016 [77]; Trinies 2015 [95]; Wall 2018 [100]; WaterAid 2009 [101]	(*n* = 6) Ismail 2016 [61]; Naeem 2015 [74]; Person 2014 [80]; Sanduvac 2017 [82]; UN Women 2017 [98]; WSSCC 2014 [102]
***Knowledge*: *Biology***	(*n* = 13) Caruso 2017 [16]; Chinyama 2019 [44]; Crichton 2013 [10]; Daniels 2016 [51]; Hemechandra 2009 [105]; Lahme 2017 [68, 69]; Mason 2013 [11]; Morrison 2016 [72]; Rheinlander 2018 [81]; Schmitt 2017 [83]; Scorgie 2016 [84]; Sommer 2009 [3, 8, 87]; Sommer 2015 [88, 89]	(*n* = 27) Al-Sabir 1998 [34]; Behera 2015 [37]; Caruso 2013 [41]; Castaneda 1996 [42]; Chothe 2014 [46]; Crawford 2014 [47]; da Silva Bretas 2011 [52]; Do Amaral 2011 [55]; Ellis 2016 [56, 57]; Garg 2001 [14]; Hosseini 2018 [60]; IWDA 2017 [62, 63]; Jewitt 2014 [64]; Long 2013 [9]; Miiro 2018 [70]; Morowatisharifabad 2018 [71]; Mumtaz 2016 [73]; Nanda 2016 [75]; Nechitilo 2016 [77]; Parker 2014 [15]; Pillitteri 2011 [79]; Secor-Turner 2016 [85]; Tegegne 2014 [93]; Titilayo 2011 [107]; Wall 2018 [100]; WaterAid 2009 [101]; Wong 2011 [109]	(*n* = 13) Dhingra 2009 [54]; Dolan 2014 [4]; Ismail 2016 [61]; Kyomugisha 1999 [67]; Naeem 2015 [74]; Narayan 2001 [76]; Person 2014 [80]; Singh 2006 [86]; Tamiru 2015 [91]; Ullrich 1992 [96]; UN Women 2017 [98]; Wall 2016 [99]; Walraven 2002 [108]
***Knowledge*: *Reproduction***	(*n* = 8) Chebii 2018 [43]; Girod 2017 [58]; Mason 2013 [11]; Rheinlander 2018 [81]; Scorgie 2016 [84]; Sommer 2009 [3, 8, 87]; Sommer 2015 [88, 89]; Sosa-Sanchez [90]	(*n* = 15) Adegbayi 2017 [32]; Caruso 2013 [41]; Castaneda 1996 [42]; Chothe 2014 [46]; da Silva Bretas 2011 [52]; Garg 2001 [14]; Guerry 2013 [59]; Jewitt 2014 [64]; Long 2013 [9]; Nanda 2016 [75]; Nechitilo 2016 [77]; Padmanadbhanunni 2017 [78]; Secor-Turner 2016 [85]; Trinies 2015 [95]; Wall 2018 [100]	(*n* = 3) Thakur 2014 [94]; UN Women 2017 [98]; Wall 2016 [99]
***Knowledge*: *Physical management***	(*n* = 9) Daniels 2016 [51]; Lahme 2017 [68, 69]; Morrison 2016 [72]; Rheinlander 2018 [81]; Schmitt 2017 [83]; Scorgie 2016 [84]; Sommer 2009 [3, 8, 87]; Sommer 2015 [88, 89]; Sosa-Sanchez [90]	(*n* = 15) Adegbayi 2017 [32]; Bilandi 2015 [38]; Caruso 2013 [41]; Chothe 2014 [46]; Do Amaral 2011 [55]; Ellis 2016 [56, 57]; Garg 2001 [14]; Guerry 2013 [59]; Long 2013 [9]; Miiro 2018 [70]; Mumtaz 2016 [73]; Tegegne 2014 [93]; Titilayo 2011 [107]; Trinies 2015 [95]; Wong 2011 [109]	(*n* = 6) Dolan 2014 [4]; Krishnan 2016 [66]; Person 2014 [80]; Sanduvac 2017 [82]; Thakur 2014 [94]; UN Women 2017 [98]
***Knowledge*: *Taboos***	(*n* = 12) Aziato 2014 [103, 104]; Caruso 2017 [16]; Chebii 2018 [43]; Chinyama 2019 [44]; Daniels 2016 [51]; Girod 2017 [58]; Hemechandra 2009 [105]; Lahme 2017 [68, 69]; Morrison 2016 [72]; Schmitt 2017 [83]; Sommer 2009 [3, 8, 87]; Sommer 2015 [88, 89]	(*n* = 24) Al-Sabir 1998 [34]; Behera 2015 [37]; Bilandi 2015 [38]; Caruso 2013 [41]; Castaneda 1996 [42]; Chothe 2014 [46]; Crawford 2014 [47]; da Silva Bretas 2011 [52]; Ellis 2016 [56, 57]; Garg 2001 [14]; Guerry 2013 [59]; IWDA 2017 [62, 63]; Long 2013 [9]; Miiro 2018 [70]; Morowatisharifabad 2018 [71]; Mumtaz 2016 [73]; Padmanadbhanunni 2017 [78]; Parker 2014 [15]; Pillitteri 2011 [79]; Tegegne 2014 [93]; Trinies 2015 [95]; Wall 2018 [100]; WaterAid 2009 [101]; Wong 2011 [109]	(*n* = 11) Amatya 2018 [36]; Dhingra 2009 [54]; Ismail 2016 [61]; Naeem 2015 [74]; Narayan 2001 [76]; Person 2014 [80]; Singh 2006 [86]; Tamiru 2015 [91]; Ullrich 1992 [96]; Umeora 2008 [97]; WSSCC 2014 [102]
**Resource Limitations**			
***Physical environment***	(*n* = 13) Budhathoki 2018 [40]; Caruso 2017 [16]; Chebii 2018 [43]; Chinyama 2019 [44]; Daniels 2016 [51]; Girod 2017 [58]; Lahme 2016 [68, 69]; Morrison 2016 [72]; Rheinlander 2018 [81]; Schmitt 2017 [83]; Scorgie 2016 [84]; Sommer 2009 [3, 8, 87]; Sommer 2015 [88, 89]	(*n* = 17) Bilandi 2015 [38]; Caruso 2013 [41]; Devnarain 2011 [53]; Ellis 2016 [56, 57]; Guerry 2013 [59]; IWDA 2017 [62, 63]; Jewitt 2014 [64]; Long 2013 [9]; McMahon 2011 [5]; Miiro 2018 [70]; Mumtaz 2016 [73]; Nechitilo 2016 [77]; Parker 2014 [15]; Pillitteri 2011 [79]; Tegegne 2014 [93]; Trinies 2015 [95]; Wall 2018 [100]; WaterAid 2009 [101]	(*n* = 3) Al-Shurbji 2017 [35]; Krishnan 2016 [66]; Sanduvac 2017 [82]; UN Women 2017 [98]
***Economic environment***	(*n* = 17) Aziato 2014 [103, 104]; Budhathoki 2018 [40]; Caruso 2017 [16]; Chebii 2018 [43]; Chinyama 2019 [44]; Crichton 2013 [10]; Daniels 2016 [51]; Girod 2017 [58]; Lahme 2016 [68, 69]; Mason 2013 [11]; Morrison 2016 [72]; Rheinlander 2018 [81]; Schmitt 2017 [83]; Scorgie 2016 [84]; Secor-Turner 2016 [85]; Sommer 2009 [3, 8, 87]; Sommer 2015 [88, 89]	(*n* = 15) Al-Sabir 1998 [34]; Behera 2015 [37]; Caruso 2013 [41]; Ellis 2016 [56, 57]; IWDA 2017 [62, 63]; Jewitt 2014 [64]; Long 2013 [9]; McMahon 2011 [5]; Miiro 2018 [70]; Parker 2014 [15]; Pillitteri 2011 [79]; Tegegne 2014 [93]; Wall 2018 [100]; WaterAid 2009 [101]; Wong 2011 [109]	(*n* = 8) Al-Shurbji 2017 [35]; Dolan 2014 [4]; Kansal 2016 [65]; Krishnan 2016 [66]; Naeem 2015 [74]; Person 2014 [80]; Tamiru 2015 [91]; UN Women 2017 [98]
**EXPERIENCE**			
***Shame and distress***	(*n* = 18) Aziato 2014 [103, 104]; Budhathoki 2018 [40]; Caruso 2017 [16]; Chebii 2018 [43]; Chinyama 2019 [44]; Crichton 2013 [10]; Daniels 2016 [51]; Girod 2017 [58]; Hemachandra 2009 [105]; Lahme 2017 [68, 69]; Mason 2013 [11]; Morrison 2016 [72]; Rheinlander 2018 [81]; Schmitt 2017 [83]; Scorgie 2016 [84]; Sommer 2009 [3, 8, 87]; Sommer 2015 [88, 89]; Sosa-Sanchez [90]	(*n* = 32) Adegbayi 2017 [32]; Al Omari 2016 [33]; Al-Sabir 1998 [34]; Behera 2015 [37]; Bilandi 2015 [38]; Caruso 2013 [41]; Crawford 2014 [47]; da Silva Bretas 2011 [52]; Do Amaral 2011 [55]; Ellis 2016 [56, 57]; Garg 2001 [14]; Guerry 2013 [59]; Hosseini 2018 [60]; IWDA 2017 [62, 63]; Jewitt 2014 [64]; Long 2013 [9]; McMahon 2011 [5]; Miiro 2018 [70]; Morowatisharifabad 2018 [71]; Mumtaz 2016 [73]; Nanda 2016 [75]; Nechitilo 2016 [77]; Padmanadbhanunni 2017 [78]; Parker 2014 [15]; Pillitteri 2011 [79]; Secor-Turner 2016 [85]; Tegegne 2014 [93]; Titilayo 2011 [107]; Trinies 2015 [95]; Wall 2018 [100]; WaterAid 2009 [101]	(*n* = 11) Al-Shurbji 2017 [35]; Amatya 2018 [36]; Boosey 2014 [39]; Dolan 2014 [4]; Kansal 2016 [65]; Kavitha 2014 [106]; Kyomugisha 1999 [67]; Naeem 2015 [74]; Narayan 2001 [76]; Ullrich 1992 [96]; WSSCC 2014 [102]
***Confidence*: *To manage menstruation***	(*n* = 6) Aziato 2014 [103, 104]; Crichton 2013 [10]; Daniels 2016 [51]; Girod 2017 [58]; Morrison 2016 [72]; Scorgie 2016 [84]	(*n* = 10) Al-Sabir 1998 [34]; Bilandi 2015 [38]; Caruso 2013 [41]; Ellis 2016 [56, 57]; Jewitt 2014 [64]; Miiro 2018 [70]; Nanda 2016 [75]; Parker 2014 [15]; Pillitteri 2011 [79]; Trinies 2015 [95]	(*n* = 0)
***Confidence*: *To engage in other activities while menstruating***	(*n* = 4) Chebii 2018 [43]; Chinyama 2019 [44]; Crichton 2013 [10]; Scorgie 2016 [84]	(*n* = 6) Al-Sabir 1998 [34]; Crawford 2014 [47]; Ellis 2016 [56, 57]; IWDA 2017 [62, 63]; Jewitt 2014 [64]; Parker 2014 [15]	(*n* = 1) Dolan 2014 [4]
***Perceptions of environments***	(*n* = 14) Budhathoki 2018 [40]; Caruso 2017 [16]; Chinyama 2019 [44]; Crichton 2013 [10]; Daniels 2016 [51]; Girod 2017 [58]; Lahme 2016 [68, 69]; Mason 2013 [11]; Morrison 2016 [72]; Rheinlander 2018 [81]; Schmitt 2017 [83]; Scorgie 2016 [84]; Sommer 2009 [3, 8, 87]; Sommer 2015 [88, 89]	(*n* = 13) Caruso 2013 [41]; Denarian 2011 [53]; Ellis 2016 [56, 57]; Garg 2001 [14]; IWDA 2017 [62, 63]; Jewitt 2014 [64]; Long 2013 [9]; McMahon 2011 [5]; Nechitilo 2016 [77]; Parker 2014 [15]; Pillitteri 2011 [79]; Tegegne 2014 [93]; Trinies 2015 [95]	(*n* = 7) Al-Shurbji 2017 [35]; Krishnan 2016 [66]; Person 2014 [80]; Sanduvac 2017 [82]; Tamiru 2015 [91]; UN Women 2017 [98]; WSSCC 2014 [102]
***Perceptions of practices***	(*n* = 13) Budhathoki 2018 [40]; Caruso 2017 [16]; Chebii 2018 [43]; Chinyama 2019 [44]; Crichton 2013 [10]; Daniels 2016 [51]; Girod 2017 [58]; Mason 2013 [11]; Morrison 2016 [72]; Schmitt 2017 [83]; Scorgie 2016 [84]; Sommer 2009 [3, 8, 87]; Sommer 2015 [88, 89]	(*n* = 16) Al-Sabir 1998 [34]; Behera 2015 [37]; Caruso 2013 [41]; Ellis 2016 [56, 57]; Garg 2001 [14]; IWDA 2017 [62, 63]; Jewitt 2014 [64]; Long 2013 [9]; McMahon 2011 [5]; Morowatisharifabad 2018 [71]; Nanda 2016 [75]; Parker 2014 [15]; Pillitteri 2011 [79]; Trinies 2015 [95]; Wall 2018 [100]; WaterAid 2009 [101]	(*n* = 12) Al-Shurbji 2017 [35]; Ismail 2016 [61]; Kansal 2016 [65]; Krishnan 2016 [66]; Kyomugisha 1999 [67]; Naeem 2015 [74]; Singh 2006 [86]; Tamiru 2015 [91]; Thakur 2014 [94]; Umeora 2008 [97]; UN Women 2017 [98]; WSSCC 2014 [102]
***Menstrual practices***	(*n* = 15) Budhathoki 2018 [40]; Caruso 2017 [16]; Chebii 2018 [43]; Chinyama 2019 [44]; Crichton 2013 [10]; Daniels 2016 [51]; Girod 2017 [58]; Lahme 2016 [68, 69]; Mason 2013 [11]; Rheinlander 2018 [81]; Schmitt 2017 [83]; Scorgie 2016 [84]; Sommer 2009 [3, 8, 87]; Sommer 2015 [88, 89]; Sosa-Sanchez	(*n* = 22) Adegbayi 2017 [32]; Al-Sabir 1998 [34]; Behera 2015 [37]; Caruso 2013 [41]; Devnarian 2011 [53]; da Silva Bretas 2011 [52]; Ellis 2016 [56, 57]; Garg 2001 [14]; Guerry 2013 [59]; IWDA 2017 [62, 63]; Jewitt 2014 [64]; Long 2013 [9]; McMahon 2011 [5]; Miiro 2018 [70]; Nanda 2016 [75]; Parker 2014 [15]; Pillitteri 2011 [79]; Secor-Turner 2016 [85]; Tegegne 2014 [93]; Trinies 2015 [95]; Wall 2018 [100]; WaterAid 2009 [101]	(*n* = 10) Al-Shurbji 2017 [35]; Dolan 2014 [4]; Kansal 2016 [65]; Krishnan 2016 [66]; Naeem 2015 [74]; Narayan 2001 [76]; Tamiru 2015 [91]; Thakur 2014 [94]; Umeora 2008 [97]; UN Women 2017 [98]
***Containment***	(*n* = 7) Chebii 2018 [43]; Chinyama 2019 [44]; Crichton 2013 [10]; Daniels 2016 [51]; Girod 2017 [58]; Mason 2013 [11]; Morrison 2016 [72]	(*n* = 16) Caruso 2013 [41]; Ellis 2016 [56, 57]; Guerry 2013 [59]; IWDA 2017 [62, 63]; Jewitt 2014 [64]; Long 2013 [9]; McMahon 2011 [5]; Miiro 2018 [70]; Morowatisharifabad 2018 [71]; Nanda 2016 [75]; Nechitilo 2016 [77]; Parker 2014 [15]; Pillitteri 2011 [79]; Trinies 2015 [95]; Wall 2018 [100]; WaterAid 2009 [101]	(*n* = 4) Boosey 2014 [39]; Dolan 2014 [4]; Naeem 2015 [74]; Tamiru 2015 [91]
***Individual menstrual factors***	(*n* = 10) Aziato 2014 [103, 104]; Chebii 2018 [43]; Crichton 2013 [10]; Daniels 2016 [51]; Hemachandra 2009 [105]; Mason 2013 [11]; Scorgie 2016 [84]; Sommer 2009 [3, 8]; Sommer 2015 [88]; Sosa-Sanchez [90]	(*n* = 22) Adegbayi 2017 [32]; Al Sabir 1998 [34]; Behera 2015 [37]; Caruso 2013 [41]; Castaneda 1996 [42]; Chothe 2014 [46]; Guerry 2013 [59]; Hosseini 2018 [60]; IWDA 2017 [62, 63]; Jewitt 2014 [64]; Long 2013 [9]; Miiro 2018 [70]; Morowatisharifabad 2018 [71]; Mumtaz 2016 [73]; Nanda 2016 [75]; Nechitilo 2016 [77]; Pillitteri 2011 [79]; Secor-Turner 2016 [85]; Titilayo 2009 [107]; Wall 2018 [100]; WaterAid 2009 [101]; Wong 2011 [109]	(*n* = 4) Kavitha 2014 [106]; Kyomugisha 1999 [67]; Naeem 2015 [74]; Walraven 2002 [108]
**IMPACTS**			
***Social participation***	(*n* = 5) Aziato 2014 [104]; Chebii 2018 [43]; Chinyama 2019 [44, 45]; Daniels 2016 [51]; Hemachandra 2009 [105]	(*n* = 15) Bilandi 2015 [38]; Caruso 2013 [41]; Crawford 2014 [47]; Garg 2001 [14]; Guerry 2013 [59]; IWDA 2017 [62, 63]; Jewitt 2014 [64]; Long 2013 [9]; Miiro 2018 [70]; Nechitilo 2016 [77]; Parker 2014 [15]; Secor-Turner 2016 [85]; Titilayo 2009 [107]; Wall 2018 [100]; Wong 2011 [109]	(*n* = 6) Al-Shurbji 2017 [35]; Amatya 2018 [36]; Krishnan 2016 [66]; Naeem 2015 [74]; Umeora 2008 [97]; UN Women 2017 [98]
***Education/employment***	(*n* = 9) Aziato 2014 [104]; Chinyama 2019 [44]; Crichton 2013 [10]; Daniels 2016 [51]; Girod 2017 [58]; Lahme 2016 [68, 69]; Mason 2013 [11]; Morrison 2016 [72]; Sommer 2009 [3, 8]	(*n* = 19) Behera 2015 [37]; Caruso 2013 [41]; Devnarian 2011 [53]; Ellis 2016 [56, 57]; Guerry 2013 [59]; IWDA 2017 [62, 63]; Jewitt 2014 [64]; Long 2013 [9]; McMahon 2011 [5]; Miiro 2018 [70]; Nanda 2016 [75]; Nechitilo 2016 [77]; Parker 2014 [15]; Pillitteri 2011 [79]; Secor-Turner 2016 [85]; Tegegne 2014 [93]; Titilayo 2009 [107]; Wall 2018 [100]; WaterAid 2009 [101]	(*n* = 8) Boosey 2014 [39]; Kavitha 2014 [106]; Kyomugisha 1999 [67]; Naeem 2015 [74]; Person 2014 [80]; Tamiru 2015 [91]; Thakur 2014 [94]; UN Women 2017 [98]
***Psychological health***	(*n* = 8) Aziato 2014 [103]; Chebii 2018 [43]; Chinyama 2019 [44]; Crichton 2013 [10]; Girod 2017 [58]; Lahme 2016 [69]; Morrison 2016 [72]; Sommer 2015 [89]	(*n* = 5) Bilandi 2015 [38]; Crawford 2014 [47]; Long 2013 [9]; Tegegne 2014 [93]; Titilayo 2009 [107]	(*n* = 5) Al-Shurbji 2017 [35]; Amatya 2018 [36]; Dolan 2014 [4]; Ullrich 1992 [96]; UN Women 2017 [98]
***Physical health***	(*n* = 11) Aziato 2014 [103, 104]; Caruso 2017 [16]; Chinyama 2019 [44]; Crichton 2013 [10]; Daniels 2016 [51]; Girod 2017 [58]; Lahme 2016 [68, 69]; Mason 2013 [11]; Morrison 2016 [72]; Rheinlander 2018 [81]; Scorgie 2016 [84]	(*n* = 7) Behera 2015 [37]; Bilandi 2015 [38]; Caruso 2013 [41]; Ellis 2016 [56, 57]; Long 2013 [9]; Nanda 2016 [75]; Parker 2014 [15]	(*n* = 3) Dolan 2014 [4]; Naeem 2015 [74]; Thakur 2014 [94]

### Antecedents of menstrual experience

#### Sociocultural context

Across cultures, menstruation was stigmatised, constructed as something that was ‘dirty’ or ‘impure’ and shrouded in silence. Menstrual stigma was a pervasive influence on women and girls. It meant that menstrual topics were not openly discussed, making it difficult to access accurate information or seek support. Negative attitudes to menstruation were internalised and manifested across multiple themes represented in the model, including as self-imposed expectations to keep menstrual status hidden, and shame and distress at the potential of having menses exposed.

Restrictive gender norms also underlay the knowledge, behavioural expectations, and social support that influenced experiences of menstruation. Expectations of female propriety and the roles of women and girls over the life course as daughters, wives, and mothers restricted resource access and proscribed care-giving tasks and movement outside the home, interacting with more immediate contributors to menstrual experience.

Proximal antecedents of menstrual experience of knowledge, social support, and behavioural expectations are described below, with supporting quotations presented in Box 1 and contributing citations highlighted in Table 3.

**Box 1. Illustrative quotations for proximal sociocultural antecedents of menstrual experience**

**Knowledge**

‘I was so scared actually I thought it was a disease that had attacked me. I thought I was going to lose my life at any moment because it was really scary to start bleeding from down there. I thought I was going to die’ (participant; Miiro 2018) [70].

‘My mother told me I would die if I showed anybody the blood’ (participant; Pillitteri 2011) [79].

‘When I first started menstruating I was shocked because I had not learned about it before…I was too embarrassed to tell my parents because I knew that they would not accept me and would say that I had bad behaviour. I could not tell them because they would say that I shamed the family and would shout at me’ (participant; Sommer 2015) [88].

‘Some people say witches can harm you if they come across (your) menstrual blood…you have prolonged periods or other complications…they can make you sterile’ (participant; Lahme 2016) [68].

Girl 1: ‘[The teacher says] you stop playing with boys on your period’. Girl 2: ‘On your period, you can get pregnant’ (participant; FGD, School 3, Private).

‘When someone is continuing with the period and is raped is there any chance to get pregnant?’ (participant; School 4, Public School) (Girod 2017) [58].

‘A few girls had received quite a lot of information from family members before they began menstruating, and they felt very confident in seeking help and managing menarche: “Both of my sisters had menstruation before than me that is why it was not difficult”‘ (Morrison 2016) [72].

‘Then my grandmother gave me one of my mother’s pads; she wasn’t able to teach me how to use it because when she was a girl she used rags only. I put the adhesive side [of the pad] on my body, instead of on my panties. I had climbed a tree with my brothers and [when I saw that blood] I thought I had hurt myself on a branch. Then, I applied alcohol [on my genitals] and I jumped!’ (participant; do Amaral, 2011) [55].

**Social support**

‘I worry who to tell because there are some mothers who when told about periods don’t care and you are forced to go and tell someone else about it to assist you [with sanitary pads]’ (participant; Crichton 2013) [10].

‘When asked about current sources of information and support on menstruation, participants said that they preferred to approach a female relative or close friend rather than a health worker. Nurses were described as “too busy” to address women’s concerns, difficult to talk to, and likely to provide only superficial and inadequate explanations”‘ (Scorgie 2016) [84].

‘In some schools, when girls go to the nurses to complain, the nurses will just tell them it is not a disease and that they should go back to their work or their school…’ (teacher; Sommer 2009) [88].

‘[I talk to] friends, [and] they help to explain about how to use sanitary pad properly, product of hygiene, and give sanitary pad because they understand about the issue and consider me their best friend’ (participant; Daniels 2016) [51].

‘Girls and teachers said that boys regularly harass girls. Girls in one focus group described multiple occasions in which boys knew that a girl had a blood stain on her uniform; the boys found ways to make the girl stand up to expose the stain’ (Girod 2017) [58].

‘You know, especially the male teachers, I don’t think they understand that when you mess yourself up it is by accident’ (participant; Lahme 2016) [68, 69].

‘The teacher had taught and told us, in the gender clubs, to use sanitary napkins. However, since male students follow us while we change menstrual soak up [absorbent] in school and teased at us, we didn’t use sanitary napkins’ (FGD participant; Tegegne 2014) [93].

‘I wore a traditional dress and my aunts sat down in a circle with me and explained to me that now I am of age and that they loved me and now I was a woman. I felt very privileged, I felt connected to them’ (participant; Padmanabhanunni 2017) [78].

**Behavioural expectations**

‘You lose some of your old friends … you should not play or mix with girls who have not started menstruating. You are not allowed to wear short dresses, skirts, or trousers’ (participant; Lahme 2016) [69].

‘[My] father plays with me when I have not experienced menstruation, but when I experience, he does not allow me to follow him’ (participant; Daniels 2016) [51].

‘During menstruation we do not have sex. If a man even touches a menstruating women he becomes ritually impure. Since both the partners are releasing heat from their bodies, if they mix, it will harm the body’ (participant; Garg 2001) [14].

‘I have heard from nearly everyone; teacher, mother, aunt and grandmother, that during my period, I should not eat cold foods, foods that produce gas, pickles, yogurt and milk because they cause stomach-ache. But I have not experienced it because I have not eaten them. In other words, I have always observed these suggestions. Well [smiling], if my mother sees me eat these things during my period, she will quarrel with me’ (participant; Morowatisharifabad 2018) [71].

‘Girls were instructed not to conduct sacred acts including praying, fasting, or touching holy books such as the Quran during menstruation. According to widely-accepted interpretations of hadith and other scholarly writings in Islam, menstruating girls and women are “ritually impure” due to the presence of blood. Girls may resume sacred acts after menstruation ends, following a ritual full-body cleansing (ghusl)’ (Nechitilo 2016) [77].

‘As one participant commented, “In my church, what they believe is that when a woman is on her periods she is dirty and you can’t come to church dirty”. Participants experienced these restrictions as “unfair” but feared that they would be offending their religious leaders and family if they did not adhere to these practices’ (Padmanabhanunni 2017) [78].

‘If the boy sees it, he will tell the family that you are having your menses, so if you prepare food he will not eat it’ (participant; Pillitteri 2011) [79].

‘If we wash at day time, there would be people moving around… people will look at us and will say that girl has no brains… we need a place where if we wash the cloth no one can see’ (participant; Caruso 2017) [16].

‘These fears also led to SSGs [Senior School Girls] practicing a secretive “don’t tell” policy about menstruation, particularly towards boys and male teachers out of fear of sharing something “improper”‘ (Rheinlander 2018) [81].

#### Knowledge

All studies reported an influence of knowledge about menstruation on women’s and girls’ experiences. Multiple domains of knowledge were discussed across studies, including the basic biology of menstruation, reproduction and the links between menstruation and pregnancy, practical management of menses and pain, and interpreting the accuracy of local taboos around menstruation.

Many authors stated that most of their study population had deficits in knowledge of menstrual biology [37, 44, 47, 55, 57, 68, 69, 71, 73, 77, 83], with some noting that only healthcare providers and biology teachers had a comprehensive understanding [95, 100]. In some studies, knowledge was more varied, with those who had attended secondary education or schools with more comprehensive reproductive health education having adequate knowledge [62–64, 83]. Knowledge deficits varied in content. In some studies, younger girls were unaware of their reproductive organs and that menstrual bleeding originated from this system, rather than elsewhere such as the digestive tract [9, 41]. In others, girls’ post menarche and older women linked menstruation with reproduction but had other lingering questions such as why menstrual bleeding occurred [11, 15, 44, 72, 73] or whether it was needed to rid the body of ‘dirty blood’ [10, 14, 34, 71, 72, 77, 84]. Near universally across studies, women and girls recalled experiences of intense distress and confusion if they were unaware of menstruation at menarche, with many participants reporting they thought they were sick or dying. In contrast, girls who had received information before menarche described more positive experiences. Where investigated, women and girls also lacked clarity on what constituted normal and abnormal (i.e., disordered) menstruation [47, 73, 85, 105, 109].

Even when aware of menstruation, participants often had insufficient information to understand the relationship between menstruation and reproduction. At menarche, many girls were told they were now fertile and to avoid males; however, this information was often provided without clarifying how or when girls were vulnerable to pregnancy [9, 32, 41, 58, 59, 64, 77, 84, 88]. Beliefs that menarche indicated sexual initiation prompted girls to hide their status for fear of punishment, reported in studies from Ethiopia [89, 100], Ghana [88], and Mali [95].

Girls expressed a strong desire for more practical information, including managing menses or pain relief [3, 8, 41, 57, 72, 73, 83, 88, 89, 93, 95, 103]. Practical knowledge about menstruation influenced the practices undertaken as well as individuals’ perceptions of their practices, with a lack of clarity around what was required for ‘hygiene’ causing concern.

Deficits in knowledge created confusion around the accuracy of cultural restrictions and taboos, creating distress and impacting confidence. Girls in many studies sought clarity on the accuracy of taboos such as appropriate foods or practices during menstruation. Several studies noted distress resulting from conflicting advice from different sources (e.g., parents, teachers, nongovernmental organisations [NGOs]) [3, 9, 44, 71, 73].

#### Social support

Social support, or lack thereof, strongly dictated menstrual experiences. Parents, siblings, peers, partners, and teachers were sources of information, resources, comfort, or assistance to accomplish menstrual tasks. Manifestation of mother–daughter communication was context dependent. In many settings, mothers served as the primary support and source of information for girls, whereas in others such as Ghana [4], Malawi [79], South Africa [84], and Tanzania [3], they were sometimes considered culturally inappropriate, and other female relatives such as aunts or grandmothers were expected to serve in this role. However, individuals’ access to support sources often varied more according to personal circumstances than country. Partners’ perceptions of menses contributed to women’s self-conceptualisation, resource availability, and the impact of menses on daily activities.

Friends and peers were critical: in school settings, they checked for stains, accompanied others to changing facilities, or provided emergency supplies. Teasing or harassment by female, but especially male, peers was highly distressing for adolescent girls. They reported great distress at males being aware of their menstrual status and bullying behaviours such as males teasing them if their status was revealed.

Healthcare providers such as nurses or community health workers were mentioned in only a few studies, and always as unsupportive [3, 84, 87, 101, 103, 109]. In studies of those with dysmenorrhea or menstrual disorders, some participants were embarrassed to seek help. Others reported pursuing multiple avenues such as doctors and herbalists or community healers in attempts to receive effective treatment for their pain [103–105, 109].

Teachers provided mixed support to girls during menstruation. Some were sensitive to girls’ needs and provided information or support such as emergency supplies, while others were uncomfortable or punitive if girls soiled themselves at school. Girls often felt more comfortable with female teachers than male teachers, who were viewed as less understanding and presented threats of sexual advances.

#### Behavioural expectations

Across studies, menstrual experience was impacted by internally and externally enforced behavioural expectations, i.e., expectations women and girls placed on themselves or those enforced by others.

Externally enforced behavioural expectations included explicit cultural or religious expectations of menstruating women as well as more implicit ideals of propriety and cleanliness placed on menstruating bodies. Explicit cultural restrictions varied across and within countries according to region, religion, caste, and individual family expectations. So too did the extent to which such restrictions directly impacted women’s and girls’ social participation. In the most severe cases, subpopulations in Nepal practiced Chhaupadi, ritual seclusion from the community and home during menstruation [36, 101]. In Zambia, Lahme and colleagues reported that participants experienced a single instance of seclusion during their first period that many girls found embarrassing and distressing [68]. In many settings, menstrual blood—and by extension, menstruating women—were considered ritually impure or polluting [10, 14, 16, 34, 37, 41, 43, 47, 60, 62–64, 68, 72, 77, 100, 101]. Such beliefs translated into restrictions on women’s behaviour, including not interacting or sitting with males; touching or cooking food; having contact with crops, livestock, or farming; or having sex. Adolescent girls reported distress at these changes to expected behaviour and fears that they would cause harm to themselves or others. In many religions, women and girls were banned from touching religious texts, praying, or entering places of worship during menstruation. Beliefs about the impurity or power of menstrual blood influenced management practices. Women and girls took steps to ensure that others could not see or access their disposed menstrual blood such as washing materials prior to disposal, eschewing single-use disposable sanitary pads, or wrapping used absorbents to prevent detection.

Other behavioural expectations stemmed from beliefs that different foods or activities would negatively impact women’s and girls’ health during menstruation. Hot or cold foods were feared to influence blood flow or clotting, and foods of various colour or flavour groups were feared to exacerbate menstrual pain, smell, acne, or cause illness.

Expectations were enforced by others, such as teachers or family, through instruction, discipline, or teasing. Family members enforcement of behavioural expectations such as religious or culturally proscribed menstrual restrictions greatly influenced the extent to which these were adhered to and thus women’s and girls’ menstrual experience. Less explicit taboos such as expectations of cleanliness, female propriety, and secrecy around menstruation were ‘enforced by adults in the community through signals and gestures’ [33]. Participants expressed fear of judgement from others should they fail to comply.

Women and girls internalised menstrual restrictions and stigma and sought to regulate their behaviour accordingly. This impacted confidence to engage in other activities during menstruation and added to experiences of shame because a failure to hide menses was viewed as a personal failure to maintain feminine standards or menstrual etiquette.

### Resource limitations

Resource deficits contributed to economic and physical environments that limited women’s choices for menstrual practices and shaped their experiences of menstruation. Quotations are presented in Box 2 and contributing citations in Table 3.

**Box 2. Illustrative quotations for resource limitations**

**Physical environment**

‘The toilets have no good doors, and anyone can see you when you are changing’ (participant; Crichton 2013) [10].

‘My friend does not have enough water. I saw her pants was blood stain[ed] because she did not put pad. She did not have money to buy [a] pad. [She has] no toilet at her house, and she used [the] toilet of her neighbor’ (participant; Daniels 2016) [51].

‘Showering was not an option in schools that experienced drought during the winter because schools had to prioritize water consumption and sanitation needs. For girls who rented a room with their siblings in the community, they also lacked the privacy to bathe at home’ (Long 2013) [9].

‘I want to use the bush. At the bush you can cover the blood that pours on the ground with sand, but in the public toilet you can’t’ (participant; Rheinlander 2018) [81].

‘The bucket/dustbin is a problem, we should be given an incinerator because the dustbin is a problem. It would be better to burn them than to carry them to dump in the latrine. And flush toilets are bad—we prefer latrines’ (participant; Sommer 2009) [87].

‘Some days we bleed heavily, and we need to change clothes at least 2 or 3 times during the school hours. There is no place to change and dispose the cloth—there is question of putting back those used cloth in our pockets. So we just bunk classes when we have to change the clothes’ (participant; WaterAid 2009) [101].

**Economic environment**

‘Several women…wanted disposable pads but indicated that distance to vendors or an inability to go to markets themselves limited their access’ (Caruso 2017) [16].

‘As the market is a little far away, we will get them only when we go ourselves … Will we ask men and boys to get it for us? …We have to use cloth and feel dirty to wash’ (participant; Caruso 2017) [16].

‘Poverty prevents girls from effectively managing their periods. For example, while girls often state that commercially-available pads are their preferred method for managing their periods, a lack of money inhibits them from purchasing pads and, in two instances, inhibited shopkeepers’ or kiosk owners’ ability to stock pads’ (McMahon 2011) [5].

‘Several factors contribute to the excessively high pricing of sanitary products in SI [Solomon Islands]. Wholesalers are subject to a 15% Goods Tax on all imported products. “Drugs, medicines, medicinal and surgical goods” are exempt from this tax, although sanitary products do not currently qualify for the exemption. The high cost of transport is another factor, but we also heard anecdotally that retailers add a 30%-40% mark-up on products such as these’ (IWDA 2017) [62].

‘Some people exchange sex for money. The money is used to buy pads. Maybe she is being given money then they have sexual intercourse…sometimes is good, sometimes it’s not because you need help, so you will just engage yourself into sex’ (participant; Mason 2013) [11].

#### Physical environment

Many studies focused on the relationships between menstruation and infrastructure, in particular, water and sanitation facilities. Women and girls reported requiring spaces to undertake a range of menstrual tasks, including changing menstrual materials, washing and drying menstrual materials, and cleaning their hands and bodies. Such spaces were often unavailable or poorly supported female needs.

Availability and characteristics of sanitation facilities and infrastructure varied across the locations frequented by participants, such as home, school, work, or public areas. Available facilities shaped experiences. An absence of sanitation facilities or safe spaces outside the home meant managing menstruation in these locations was more challenging, reducing confidence to travel outside the home and increasing distress and fear of shame. Characteristics of the infrastructure such as the distance to or availability of soap and water, as well as the presence of locks or lights, influenced the menstrual practices that could safely be undertaken. The availability of a bin or incinerators or community waste disposal or the use of pit latrines influenced disposal choices. There were mixed perspectives on disposal options. For women and girls, the priority was perceived privacy, with concerns that materials would attract pests. Teachers and authors expressed concerns about blockages to sanitation systems.

The way that women and girls perceived the available environments for menstrual management was an integral part of their experience, expanded upon below. This represented a combination of what was available with personal needs and expectations for cleanliness, privacy, and ‘appropriate’ behaviours. In schools, gender-separate latrines and locks on doors were frequently discussed as compromising privacy, as was a lack of water to wash hands and remove evidence of menstrual blood from latrines.

Environmental considerations were noted in studies across varied geographies. Wet seasons and weather presented challenges for thoroughly and discretely drying reusable menstrual materials [16, 79]. Water scarcity altered behaviours where this resource was limited and was therefore conserved during washing absorbents or cleaning blood from hands or latrines [88, 89, 100]. Two studies undertaken in a cold climates found that latrines were too cold to be used during winter months [35, 77].

#### Economic environment

Lack of funds to purchase menstrual items or pain relief, and lack of affordable cloth or commercial menstrual products, was a frequent study finding. Many women and girls struggled to afford their choice of material, some studies noting that the household frequently lacked money for basic necessities and that commercial sanitary pads were considered an unaffordable luxury [5, 10, 15, 41, 43, 51, 64, 68]. Adolescent girls in poor households noted that requesting money for menstrual needs could cause friction in the household [10, 11]. Girls regularly undertook paid employment to generate funds. Two studies from Kenya and one in Ghana noted concerns that girls engaged in transactional sex to meet menstrual and other personal funding needs [4, 11, 64]; authors of a study in Ethiopia noted that this was not reported in their population [100]. In some studies, authors noted that teachers and NGOs provided menstrual materials, but that this was unreliable [44, 58]. Similarly, in humanitarian contexts, participants expressed that material provision was inconsistent [15, 35, 83]. Stock shortages and inflated pricing of menstrual products due to remote locations, retailer mark-up, and taxes were noted in some studies [5, 62, 63, 104]. In humanitarian contexts, changed access to materials was salient in women’s experiences as they adapted to new management practices or were unable continue their past preferred behaviours [40, 83].

### Menstrual experience

Illustrative quotations of menstrual practices and perceptions are provided in S2 Table; Box 3 shows themes of containment, confidence, and shame.

**Box 3. Illustrative quotations for themes of containment, confidence, and shame**

**Containment**

‘Girls also stated their fear that others would know when they are on their period, that they would “smell” or “see” their stained clothes, that they would make fun of them, especially the boys. “I can be worried because in our class we have stubborn boys […] I am not comfortable that, if it comes on my clothes everyone will know”‘ (participant; Guerry 2013) [59].

‘Cloths were frequently mentioned as ineffective because blood leaks through the cloth, a bloodied cloth can slip out of panties and fall on the ground, and bloodied cloth smells “like bad eggs” or feels wet or heavy’ (McMahon 2011) [5].

‘I only have one uniform, so when I stain my skirt I go home and stay there until my skirt is dry’ (participant; Pillitteri 2011) [79].

‘To assist with menstrual management in schools, the girls recommended the need to have emergency pads, underwear, extra clothing including shawls and pain medication, be available for girls who do not have the MHM-related materials they need’ (Mumtaz 2016) [73].

**Confidence**

‘[I can manage] because [my] parents often tell [teach] me, and I clean [my] vagina well. I am able to know about menstruation and what to do for well hygiene, and [I] have enough sanitary pad[s]’ (participant; Morrison 2016) [72].

‘In the following example, feeling positive about menstruation required having access to sanitary pads and feeling confident about how to keep menstruation secret: “When I am at home, I don’t like sitting in the house. I prefer going outside when I have my pad on and I am not afraid to play with my friends”‘ (Crichton 2013) [10].

‘… You feel very uncomfortable, but madam we have no choice’ (participant; Lahme 2016) [68, 69].

‘I think it definitely shapes our perceptions of our bodies, at least it did me. I think it leads to a lot of confusion about your body and what it’s supposed to be doing, and I think it also informs our perceptions about what our role is as a wife, or a mother, or a sister, because if you have grown up in a very strict household where the ritual was very strongly emphasised, then you will go through life where you think you are untouchable for the four days of the month’ (participant; Crawford 2014) [47].

‘In PNG [Papua New Guinea] and SI [Solomon Islands], some female participants described staying home during their menstrual periods and avoiding interactions. This is often self-imposed and frequently relates to a fear of clothes being stained by menstrual blood or fear that others can smell menstrual blood’ (IWDA 2017) [63].

‘You can’t be free when you are menstruating like you always are when you are not [menstruating]. When you are menstruating and you are in class, maybe you do not sit properly and blood leaks on the pant [underwear] and out on the uniform. So you can’t be free, you will just be thinking that maybe you have messed up. So it’s better to be at home’ (participant; Chinyama 2019) [44].

‘A couple of girls said it was easier to concentrate if they knew when to expect their periods, and suggested tracking the dates on a calendar: “If may be you want to be remembering fast on your own you can write a calendar.” One remarked that it used to be more difficult to concentrate back before their schools started providing pads, saying “aah it’s better, there [are] pads, we are now moving on the same page [as] boys…”‘ (Nanda 2016) [75].

**Shame and distress**

‘Girls expressed concern that if they were to use the toilet for managing menstruation and there was a line of students, other students would then know they were menstruating because of increased time of toilet use or blood left on the seat or toilet bowl. [G]irls expressed embarrassment, shame, and anxiety related to these possibilities’ (Ellis 2016) [56].

‘Well, I have a very big family, 11 sisters and six brothers. My mother, if we’d ask any kind of question like that, she would slap our mouth; she was very severe! So I thought I was going to die; I didn’t know what it was when I bled for the first time. Even so I decided to hide it Every time I bled, I’d spend a whole day in bed because I thought I was going to die. This lasted for six months’ (participant; do Amaral 2011) [55].

‘Girls felt fear and shame in anticipation of a bloodstain, an odour, or a classmate realizing she was menstruating because these outcomes all resulted in humiliation, laughing and teasing from classmates’ (Long 2013) [9].

‘In our community we have never talked about it because we feel ashamed’ (participant; P8 Parent FGD 3) [11].

‘Blood is something so secret that it is not recommended anyone to see’ (participant; P4 School K) [11].

‘You can have fear because sometimes you live with your father, and at times you cannot share with your father these things’ (participant; P1 School B) (Mason 2013) [11].

‘This was ascribed to the assumption that men viewed menstruation as “repulsive” and “tainting” and would consider them less desirable if their menstrual status was revealed’ (Padmanabhanunni 2017) [78].

‘I enjoy my periods, they are painful but I look forward to them, they confirm that I am a woman, and every month I am reminded that I am a woman and I can bring children into the world’ (participant; Padmanabhanunni 2017) [78].

#### Menstrual practices

The behaviours undertaken to manage menstrual bleeding were a central component of the experiences reported. While studies differed in the extent to which they explored these practices, together they provided a comprehensive picture of the range of practices women and girls undertake to manage menses (S2 Table). These included accessing and using materials to absorb menses, changing materials and disposing of them, washing hands before or after changing materials, washing genitals, and washing and drying reusable materials. Transportation of clean and used materials, as well as their storage between menses, were highlighted in a smaller number of studies. Practices were strongly linked to participants’ experiences of physical health symptoms, with suggestions that unhygienic practices directly cause irritation and infection [9–11, 41, 44, 51, 56, 68, 72, 75]. Participants frequently interpreted genital irritation as resulting from properties of their menstrual materials. Many studies highlighted inadequate access to preferred, comfortable materials to absorb menses as problematic. The materials used, and the resources to undertake menstrual practices such as washing hands, were associated with women’s and girls’ levels of confidence to manage menstruation and their confidence to engage in other activities.

#### Perception of practices and environments

A critical distinction emerged between reported menstrual practices and women’s and girls’ perceptions of them, i.e., if women were able to use their preferred practices and if these were perceived as reliable and comfortable (S2 Table). Perceptions were heavily influenced by women’s and girls’ understanding of menstruation and their internalised expectations of optimal practices. Similarly, while the physical environment impacted on menstrual experience, perceptions of the environments’ cleanliness, privacy, and safety were an integral component of experience. The stigmatised nature of menstruation, taboos against the visibility of menstrual blood, and threat of sexual advances or teasing by males all contributed to women’s and girls’ perceptions of the adequacy of different environments. For example, in some cultures, even if disposal bins were available within latrines, taboos around others seeing menstrual blood meant that these were perceived as inadequate because they left women and girls feeling vulnerable to embarrassment or physical harm through witchcraft [14, 72, 84].

Participants described different levels of satisfaction with the same practices or environments. In some studies, this manifested in generational tensions between mothers and daughters, where mothers felt that daughters already had greater access to materials or knowledge than they had experienced [5, 97]. In humanitarian contexts, maintaining expected standards of cleanliness and menstrual etiquette was challenged when practices and environments had changed [35, 40]. Perceptions reflected an interaction between resources and behavioural expectations and were often more salient to confidence, shame, and psychological, and social consequences than the menstrual practices themselves.

#### Containment

Containment was fundamental to participants across populations and included leakage, keeping materials in place, and minimising detectable odour. Authors conceptualised containment challenges differently, with some viewing soiling or odour as failures of resources to meet females’ basic needs causing frustration, while others viewed concerns about visible menstrual blood as symptoms of the stigmatised nature of menstruation and enforced need for concealment. Direct quotations from participants supported both discourses. They expressed fear at stigma or bullying invoked by revealed menstrual status; at the same time, they expressed frustration at the need to clean limited supplies of cloths or linens or at the experience of odour. Similarly, cloths falling out of underwear was viewed as both embarrassing and a frustrating restriction of activities. The now-dirty materials needed to be cleaned and re-affixed. Internalised expectations of menstrual etiquette, keeping clean and discrete, meant that failures to contain menstrual blood or odour were viewed as personal failings imbued with embarrassment and distress.

#### Confidence

Women and girls reported varying levels of assurance that they were able to manage their menstruation and could undertake other activities during menstruation. These closely related subthemes were a salient part of menstrual experience. In our integrated scheme, this is titled as confidence but includes concepts of self-efficacy and perceived agency.

Women’s and girls’ confidence that they could execute the necessary tasks to manage their menses pervaded reports of menstrual experiences although was rarely identified by authors as an independent theme. Reading across studies, authors consistently noted improved confidence to manage menstruation when participants had access to preferred materials and environments, greater knowledge, and access to social support [10, 15, 34, 38, 41, 51, 56, 58, 64, 70, 72, 75, 79, 84, 95, 103, 104]. One study identified and defined this construct as menstrual self-efficacy [51]. Studies with greater interpretive depth noted that a lack of confidence to manage menses influenced how women and girls perceived themselves and their bodies, internalising a sense that ‘their bodies are beyond their control’ [5, 16, 47]. Conversely, greater confidence positively impacted outcomes.

Throughout studies, authors also reported varied levels of confidence to engage in other activities while menstruating. This was influenced by women’s and girls’ confidence that they could manage menstruation and past experiences of failed containment, as well as by the support available. In some studies, this confidence was expressed as women’s and girls’ sense of ‘freedom’ or empowerment during menstruation, that they could freely participate in social activities or travel outside the home without preoccupation with their menstruation [10, 43, 44]. Confidence to undertake other activities during menstruation was frequently the mechanism through which poorly perceived menstrual practices and shame translated into impacts on social participation and well-being.

#### Shame and distress

Across settings and studies, menstruation was overwhelmingly associated with feelings of shame and distress. Internalised menstrual stigma and expectations of silence on the topic resulted in negative attitudes and intense fears of shame should menstrual status be exposed. In most studies, some participants reported experiences of intense distress recalling incidents when menstrual status was revealed. Distress—including worry and fear—associated with menstruation were highlighted by authors as significant burdens in the lives of women and girls and as indicative of the need for more attention to the topic [3, 10, 11, 16, 40, 43, 44, 51, 68, 72, 81, 83, 84, 88, 90].

In some studies, authors noted that participants, typically a small minority, expressed a positive sentiment regarding their menstruation as affirmation of their womanhood and fertility [5, 8, 32, 43, 47, 51, 52, 55, 60, 71, 78, 85, 109]. For these women and girls, menstruation was viewed as a healthy bodily function, and the stress of management was tempered by pride in being a woman and the potential of joy of motherhood.

#### Individual menstrual factors

Physical symptoms during menstruation—particularly menstrual pain such as cramping of the abdomen, back, and legs—was a prominent part of women’s and girls’ experiences. Participants also reported fatigue, headache, nausea, vomiting, breast tenderness, skin blemishing, and irritability. Menstrual symptoms and the presence of abnormalities or disorders moderated individuals’ experiences, exacerbating shame and reducing confidence if participants felt that their experience was abnormal and making it more difficult to conceal menses. Pain directly restricted school, work, and social participation. In some studies, girls expressed a desire for a safe place to rest at school to manage menstrual cramping [8, 88]. Awareness of, and access to, pain relief methods varied. In some studies, participants reported used strategies such as paracetamol or local herbal remedies, hot water, or rest to combat menstrual pain. Individual menstrual variations, such as the extent of bleeding, influenced perceptions of adequate practices and environments. Despite being the focus of very few studies, antecedents of menstrual experience, including knowledge, social support, behavioural expectations, and the resource environment, were all relevant to managing pain and other symptoms [103–109]. Few women accessed healthcare, and the economic environment restricted access to pharmaceutical pain relief. In 3 studies, authors reported myths that pharmaceutical pain relief would result in health detriments or infertility [62, 70, 79].

### Impacts

Illustrative quotations are presented in Box 4, and a summary of contributing citations is displayed in Table 3.

**Box 4. Impacts of menstruation, illustrative quotations**

**Physical health**

‘If you don’t wash and change your sanitary pads regularly you can have rashes and bruises. It’s very common to feel itching when you are using old rags or pieces of blankets…you feel very uncomfortable’ (participant; Lahme 2016) [68].

‘If the cloth used is dirty, i.e., not cleaned properly before it is used, it can cause boils and sores to form on private parts’ (participant; Garg 2001) [14].

‘When washing the cloth the bacteria may get transferred to our hands, and it is also disgusting to wash the cloths…Later on we have used the same hands to eat and do many other things’ (participant; Morrison 2016) [72].

‘When you are using mattress it can cut into small pieces and it can get inside the vagina. [S]o she can get problem in the vagina’ (participant; P9 School D). ‘She can get cancer’ (participant; P3 School D) (Mason 2013) [11].

**Psychological health**

‘Participants used language like “feeling bad”, feeling “stressed”, or “fearful” and “wanting to cry” to describe the emotional distress they experienced’ (Crichton 2013) [10].

‘While being secluded, girls reported being bored, lonely, and depressed: “I was bored sitting there alone. I felt like no one loved me…I felt like crying…”‘ (Morrison 2016) [72].

‘During my first menstruation I was shocked and embarrassed. Generally, whenever I have it, I think that I am below humans, depressed, eh…I hate being female; I assumed it as a disease…’ (participant; Tegegne 2014) [93].

‘The girl cries, “Am I sick or what? Am I pregnant or what”…she feels bad about herself…a girl could even commit suicide. She could come to that…if it was a single girl [she asks herself], “And now what am I going to do? What am I worth?” In this situation, many poison themselves…and many times you’re not pregnant, only that the blood has stuck together, that clot…It’s in vain that they have worried’ (participant; Long 2013) [9].

**Education and employment**

‘Girls missed parts of class when they had to change menstrual hygiene materials. This was due to short breaks between classes, in combination with the long distance girls had to walk to find a private place outside to change, or if they returned to their homes to manage menstrual hygiene’ (Long 2013) [9].

‘I felt like, oh no… I didn’t want to stand up in class. My friends told me to go for lunch [with them]. I told them I didn’t want to because I was having my periods…I thought, like, I didn’t want anyone to come near me. I thought, like, they will get to know. Maybe I’m smelling…’ (participant; Miiro 2018) [70].

‘For example maybe during the exams, if [your] period starts in the exams room, they will remove you from the exams even though maybe you were on question 1, they will remove you from the exams and say go and make yourself clean, you will make the classroom to stink and then you will be disappointed and then you will start to say so I have missed my exams, so instead of you passing you will fail at the end’ (participant; Nanda 2016) [75].

‘…I never used to go to class for almost 3 days. I used to sit in the sick bay and sleep…I used to tell my teacher I am sick’ (participant; Jewitt 2014) [64].

‘In addition, if sanitation and disposal facilities at schools or in the workplace are nonexistent or not usable (i.e., not functional, lacking in privacy or filthy), women and girls prefer to go home to change materials, or delay changing materials for extended periods of time’ (IWDA 2017) [62].

**Social participation**

‘When I am menstruating, I don’t run…I don’t play around. I just stay quiet. I run and get a heavy flow. Also when I play, I get a heavy flow’ (participant; Secor-Turner 2016) [85].

‘I feel frightened to go around and meet people because of the smell, so I usually go to the garden to stay away from people in the village’ (participant; IWDA 2017) [62].

‘Since I am the eldest in the family, I had to help my father with the cattle and the crops but when I am menstruating I have to tell my mother and she passes it on to my father…he will then remind me that I mustn’t come near where he is working’ (participant; Padmanabhanunni 2017) [78].

‘Even now, I stay separately for four days. And I don’t touch food, I don’t go to the kitchen, and I don’t cook, and I don’t go to…puja [an act of prayer or worship]…religious function[s]’ (participant; Crawford 2014) [47].

#### Physical health

Women and girls reported genital discomfort, irritation, rashes, and bruising during menses stemming from the properties of menstrual materials or inadequate frequency of change [9–11, 15, 37, 41, 44, 51, 56, 57, 68, 69, 72, 75]. Authors linked these reports to reproductive tract infections, supported by key informants such as healthcare providers. In many settings, participants emphasised the importance of ‘keeping clean’ during menstruation to prevent disease and were anxious to maintain this standard. However, beliefs about the practices required for cleanliness or the associated health consequences varied. In some settings, women and girls expressed a need to wash genitals many times per day to maintain hygiene, while in others, washing during menstruation was thought to impact blood flow. In some studies, authors reported a mix of experiences of symptoms such as irritation, as well as participants’ perceived health risks. Participants reported fears of infertility or cancer resulting from unhygienic menstrual management [11, 44, 97]. In others, women and girls were concerned that they could contract genital infections or viruses from using unclean sanitation facilities [16, 56, 81, 84]. Authors identified health impacts resulting from adherence to taboos such as dietary restrictions on the consumption of common foods, linked to fatigue, or withholding urination for fear of others observing menstrual blood in latrines [16, 41, 56, 57].

#### Psychological health

Reading within and across studies, our integrated model separated the negative menstrual experiences of poor confidence to manage menstrual bleeding or shame and distress associated with menstruation, from the impacts of this experience on broader psychosocial well-being and mental health, i.e., impacts on anxiety, depression, and well-being beyond the immediate menstrual experience. Studies noted the potential for negative menstrual experiences to have broader psychological impacts [9, 10, 43, 44, 47, 68, 89, 93, 107]. Authors noted the erosion of self-esteem due to menstrual experiences, the contribution of stress during menstruation to anxiety, and depression linked to reduced social participation or experiences of stigma. Studies of those with menstrual disorders highlighted feelings of powerlessness in addressing dysmenorrhea or associated symptoms that may negatively impact psychological outcomes for these individuals [60, 103–105].

#### Education and employment

Many studies that focused on girls of school age reported consequences for education [3–5, 8–11, 15, 37, 39, 41, 44, 51, 56–59, 62–64, 67–70, 72, 75, 77, 79, 85, 87–89, 91, 93, 94, 100, 101, 104, 106, 107]. Some highlighted full- or part-day absences, and others detailed disengagement from class when girls were present. Reduced participation was triangulated with reports from teachers. Multiple aspects of menstrual experience contributed. Poorly perceived menstrual practices such as unreliable absorbents that girls felt were liable to leak or expose odour reduced attendance and engagement [8, 9, 11, 37, 39, 41, 56–59, 64, 68, 69, 72, 75, 79, 80, 85, 91, 93, 94, 100, 101]. Girls lacking knowledge and confidence to manage menstruation were reluctant to be around others [3–5, 8, 39, 70, 75, 77, 89]. Unsupportive infrastructure was problematic. In many schools, girls reported that they had no adequate location to change absorbents, feared others seeing menstrual blood in latrines, or lacked facilities to dispose or clean absorbents [4, 5, 9, 10, 15, 41, 51, 56–58, 64, 70, 72, 74, 79, 87, 88]. This meant travelling home to change menstrual materials, often resulting in part-day absences. Menstrual pain made school attendance challenging. Some studies reported that teachers would send girls home from school, or punish them, if they detected odour or soiled outer garments, directly linking enforced behavioural expectations to education [41, 43, 81]. Where menstrual restrictions forbade girls from travelling outside the home or being around males, these also directly impacted school attendance.

Fewer studies attended to the experiences of adult women, and most explored sanitation infrastructure and menstrual experiences at home rather than at work. Some studies linked social exclusion, restriction of travel, and infrastructure deficits to impacts on formal or informal employment [39, 62, 63, 98].

#### Social participation

Women and girls reported altering movement and participation outside the household during menses, as well as restricting activities such as running or playing sports [9, 14, 15, 35, 36, 38, 41, 43, 44, 47, 51, 59, 62–64, 70, 74, 77, 85, 97, 98, 100, 103–105, 107, 109]. Many motivations echoed those restricting school attendance, with women and girls experiencing pain or afraid their menstrual status would be exposed, that others would detect odour, or that menstrual materials or blood would fall out of place. Behavioural expectations in many settings also restricted interactions with males, food preparation, or participation in religious gatherings or activities. As noted above, women expressed varied adherence to and frustrations with explicit restrictions on activities during menstruation, some appreciating rest and others disliking being excluded. Some restrictions were interconnected with expectations of propriety placed upon girls as they sexually matured, to protect from sexual advances, as they could now become pregnant.

## Discussion

This systematic review had 2 overarching objectives: (1) to synthesise extant qualitative studies of women’s and girls’ menstrual experience in LMICs and (2) to integrate findings across studies to develop a directional model of menstrual experience to advance problem theory in menstrual health research. Despite different settings and populations, the narratives and lived experiences that emerged reflected consistent themes, with manifestations that differed by context. Mapping relationships between themes highlighted the multidimensional nature of menstrual experience. The integrated model produced illustrates pathways through which distal and proximal antecedents influence menstrual experience and ultimately result in impacts on physical and psychological health, education, employment, and social participation.

Metasynthesis identified multiple components of menstrual experience. Menstrual practices, such as the type of material used, represented only one of these and directly contributed to physical health outcomes alone. Women’s and girls’ perceptions of their menstrual practices and environments emerged as a critical independent theme connecting practices to other impacts such as school absenteeism. In addition, confidence to manage menstrual bleeding and to undertake other activities during menses, as well as experiences of shame and fear, were salient contributors to psychological, social, and educational outcomes. Distal antecedents—the sociocultural context and resource limitations—underlay more proximal factors. Experiences captured in qualitative studies revealed knowledge of the biological, reproductive, and practical aspects of menstruation as a source of confidence, shame, and perceived acceptability of practices and environments. Social support enhanced or diminished experiences. This was closely connected to behavioural expectations that were placed upon women and girls and were enforced through discipline and cultural practices and ideals of cleanliness or femininity. Such expectations and broader menstrual stigma were internalised, influencing experience and self-concept. Poorly supportive physical infrastructure, such as a lack of water and sanitation facilities, made it difficult for women and girls to undertake their preferred menstrual practices in privacy and safety; in addition, the economic environment restricted access to preferred materials, soap, and pain relief.

Included studies represented a broad range of contexts, although the majority attended to the experiences of school-aged girls. Amongst this group, we achieved early saturation of impacts and antecedents. Qualitative studies identified different relative contributions of antecedents such as the physical environment, knowledge, or behavioural expectations (particularly explicit restrictions) for girls in different settings, which have implications for programming in those areas. However, researchers should reconsider burdening future populations to identify these same factors and should focus on more detailed research questions. Greater depth in understanding the transmission of social norms and behavioural expectations, as well as confidence during menstruation, requires more attention. Experiences of adult women, particularly in the workplace, were under-researched. The experience of pain and menstrual disorders was poorly integrated in included studies and did not reach saturation. Most studies relied on FGDs. This approach was often selected to source priorities for intervention but meant that studies more often captured shared experiences of the school environment, rather than deep understanding of individual experiences that may yield greater sociological or psychological insights.

### Strengths and limitations

The model presented differs from previous nonsystematic summaries that have generated lists of considerations to improve menstrual experience but have not specified directional relationships. Systematic searching identified a large body of literature, facilitating clarity in the identified themes. Metasynthesis remains an evolving methodology for the review of qualitative literature [30, 110–112]. This work draws on current best practice guidance [26, 27, 29, 30, 112]. As in all reviews, the findings are limited by available studies. The majority of included studies came from sub-Saharan Africa, and findings may be more representative of this context. We identified no studies from China, and studies from the Middle East and North African countries were more frequently undertaken in urban, more highly educated populations, though this reflects the global focus on menstrual hygiene research in specific geographical areas. Despite extensive searching, eligible studies may have been missed. A small number of studies in languages other than English were included; however, searching was only undertaken in English and may not have identified all available publications. Study quality was appraised using a tool designed for qualitative studies. This may have resulted in poorer scoring of mixed-methods studies that were considered only based on their qualitative components. Finally, as with any systematic review of qualitative research, a different team of researchers may have generated different insights. However, our focus on consensus themes and auditable methods lends credibility to our results as presented.

This review included a large volume of studies. This presented the opportunity to draw themes and an integrated model across a broad range of contexts and populations. Region-specific reviews, or reviews with more targeted research questions, may have space to provide deeper insights into specific questions or region-specific challenges.

### Implications for research and practice

Findings of our synthesis have implications for research and practice. To date, developed interventions have sought to improve menstrual experience by investing in education (to improve knowledge) and menstrual products (to improve the economic environment), two of the core pillars of existing menstrual health frameworks [19, 113]. Social support, the physical environment, cultural restrictions, and the perception of menses as dirty and needing to be concealed were all highly salient in included studies, yet these risk factors have received limited attention in interventions. Furthermore, these factors likely represent important covariates that should be considered when not the focus of interventions. The integrated model presented here offers a more nuanced framework to inform theories of change in program development and to assist evaluation strategies.

Awareness of multiple, interconnected constructs also aids the identification of unanticipated harms. Models focused exclusively on menstrual practices have assessed only physical harms such as infections. Our integrated model suggests that changes to girls’ perceptions of their practices may cause distress as they struggle to adopt new practices advised in education interventions in an unsupportive physical environment. Interventions that focus on products may maintain menstrual stigma and reinforce behavioural expectations that concealing menses is paramount. Such interventions may have positive impacts on containment in the short term but risk greater harms if access to more reliable menstrual materials is unsustained.

Results have implications for the design of quantitative studies. To date, these have evaluated links between menstrual practices and impacts (e.g., sanitary pad use and school attendance) [18, 114]. Our integrated model suggests that this is an indirect relationship and is unlikely to capture the true effects of menstrual experience on psychological, educational, and social outcomes without attention to the other components of experience that mediate this relationship. This integrated model should inform hypotheses for more detailed quantitative studies to test the pathways emerging from qualitative research and subsequently inform intervention research. Furthermore, it may serve to inform measure development for identified components of menstrual experience, antecedents, and impacts.

The definition ‘menstrual hygiene’ outlined in the Introduction fit poorly with the integrated model resulting from this metasynthesis. This definition provides an unclear list of menstrual practices, incorporates one mention of perceptions of the menstrual environment but not practices, and refers to physical facilities for disposal only. Recent expansions on this definition to include menstrual knowledge conflates menstrual experience with antecedents [115]. As presently defined, ‘menstrual hygiene’ may reflect an incomplete set of menstrual rights or needs, or it could be amended to capture menstrual practices as have emerged here. Findings of this review suggest that terminology and construct definition in menstrual health research may need to be expanded to recognise the many components of menstrual experience and contributing factors.

### Conclusions

In sum, extant qualitative studies have identified consistent negative impacts for health and social participation resulting from poor menstrual experience. This large body of qualitative evidence emphasises the need for practitioners and policymakers to attend to menstruation to improve the physical and psychological health, educational attainment, and social participation of women and girls. The integrated model presented advances the development of problem theory in menstrual health research and highlights important factors to consider in future research and practice. Menstrual experience is characterised not only as the hygiene practices undertaken to manage menstrual bleeding but by women’s and girls’ perceptions of these practices, their confidence to manage menses and engage in other activities while menstruating, and their experience of shame and containment. Through synthesis, we elucidate antecedent pathways and highlight the multiple components of menstrual experience that must be considered for effective interventions and comprehensive quantitative evaluation.

## Supporting information

S1 PRISMA Checklist PRISMA, Preferred Reporting Items for Systematic Reviews and Meta-Analyses. (PDF)

S1 Text Grey literature searching: List of organisation websites. (PDF)

S1 Table Study quality appraisal using the EPPI-Centre checklist, with brief notes regarding the rating assigned. (PDF)

S2 Table Illustrative quotations for themes of menstrual practices, perceptions of menstrual practices, and perceptions of the environments in which practices are undertaken. (PDF)

## Abbreviations

FGD: focus group discussion
LMIC: low- and middle-income country
MHM: menstrual hygiene management
NGO: nongovernmental organisation
PRISMA: Preferred Reporting Items for Systematic Reviews and Meta-Analyses
