# Supplementary material for: Women’s and girls’ experiences of menstruation in low- and middle-income countries: A systematic review and qualitative metasynthesis
Source: PLoS Med. 2019 May 16;16(5):e1002803. doi: 10.1371/journal.pmed.1002803 (PMC6521998; doi:10.1371/journal.pmed.1002803)
Supplement: S1 PRISMA Checklist — (PDF) [file pmed.1002803.s001.pdf]

PRISMA 2009 Checklist

Section/topic	#	Checklist item	Reported: (p=paragraph no)
**TITLE**			
Title	1	Identify the report as a systematic review, meta-analysis, or both.	Title.
**ABSTRACT**			
Structured summary	2	Provide a structured summary including, as applicable: background; objectives; data sources; study eligibility criteria, participants, and interventions; study appraisal and synthesis methods; results; limitations; conclusions and implications of key findings; systematic review registration number.	Abstract.
**INTRODUCTION**			
Rationale	3	Describe the rationale for the review in the context of what is already known.	Background.<br>P1 (MH experience)<br>P2 (lack of synthesis)<br>P3-4 (lack of problem theory)
Objectives	4	Provide an explicit statement of questions being addressed with reference to participants, interventions, comparisons, outcomes, and study design (PICOS).	Background P5, Abstract
**METHODS**			
Protocol and registration	5	Indicate if a review protocol exists, if and where it can be accessed (e.g., Web address), and, if available, provide registration information including registration number.	Abstract,<br>Methods P1
Eligibility criteria	6	Specify study characteristics (e.g., PICOS, length of follow-up) and report characteristics (e.g., years considered, language, publication status) used as criteria for eligibility, giving rationale.	Methods: Searching and Selection: P2
Information sources	7	Describe all information sources (e.g., databases with dates of coverage, contact with study authors to identify additional studies) in the search and date last searched.	Methods: searching and selection: P1
Search	8	Present full electronic search strategy for at least one database, including any limits used, such that it could be repeated.	Methods: Box 1
Study selection	9	State the process for selecting studies (i.e., screening, eligibility, included in systematic review, and, if applicable, included in the meta-analysis).	Methods: Searching and Selection: P2
Data collection process	10	Describe method of data extraction from reports (e.g., piloted forms, independently, in duplicate) and any processes for obtaining and confirming data from investigators.	Methods: Searching and Selection : P3;
Data items	11	List and define all variables for which data were sought (e.g., PICOS, funding sources) and any assumptions and simplifications made.	Study characteristics reported Table 1.<br>Data was qualitative reports. Analysis described Methods: Analysis.

Risk of bias in individual studies	12	Describe methods used for assessing risk of bias of individual studies (including specification of whether this was done at the study or outcome level), and how this information is to be used in any data synthesis.	Methods: Study quality appraisal.
Summary measures	13	State the principal summary measures (e.g., risk ratio, difference in means).	Methods: Analysis P1 and 2
Synthesis of results	14	Describe the methods of handling data and combining results of studies, if done, including measures of consistency (e.g., I^2) for each meta-analysis.	Methods: Analysis P1 and 2

Section/topic	#	Checklist item	Reported on page #
Risk of bias across studies	15	Specify any assessment of risk of bias that may affect the cumulative evidence (e.g., publication bias, selective reporting within studies).	Qualitative review. Bias considered in analytic strategy.<br>Results: P3; Table 1 and S1 Table. Table 2.
Additional analyses	16	Describe methods of additional analyses (e.g., sensitivity or subgroup analyses, meta-regression), if done, indicating which were pre-specified.	Methods: Analysis: P2
**RESULTS**			
Study selection	17	Give numbers of studies screened, assessed for eligibility, and included in the review, with reasons for exclusions at each stage, ideally with a flow diagram.	Fig 1.
Study characteristics	18	For each study, present characteristics for which data were extracted (e.g., study size, PICOS, follow-up period) and provide the citations.	Table 1.
Risk of bias within studies	19	Present data on risk of bias of each study and, if available, any outcome level assessment (see item 12).	Table 1 + S1 Table.<br>Table 2 – outcome level assessment.
Results of individual studies	20	For all outcomes considered (benefits or harms), present, for each study: (a) simple summary data for each intervention group (b) effect estimates and confidence intervals, ideally with a forest plot.	NA
Synthesis of results	21	Present results of each meta-analysis done, including confidence intervals and measures of consistency.	NA (integrated scheme presented Fig 2)
Risk of bias across studies	22	Present results of any assessment of risk of bias across studies (see Item 15).	Table 1; Supplementary Materials 2,
Additional analysis	23	Give results of additional analyses, if done (e.g., sensitivity or subgroup analyses, meta-regression [see Item 16]).	NA
**DISCUSSION**			
Summary of evidence	24	Summarize the main findings including the strength of evidence for each main outcome; consider their relevance to key groups (e.g., healthcare providers, users, and policy makers).	Discussion: P1-3

Limitations	25	Discuss limitations at study and outcome level (e.g., risk of bias), and at review-level (e.g., incomplete retrieval of identified research, reporting bias).	Discussion: Strengths and limitations
Conclusions	26	Provide a general interpretation of the results in the context of other evidence, and implications for future research.	Discussion: Implications for research and practice
**FUNDING**			
Funding	27	Describe sources of funding for the systematic review and other support (e.g., supply of data); role of funders for the systematic review.	Journal system.<br>Sections:<br>Financial disclosure Statement

From: Moher D, Liberati A, Tetzlaff J, Altman DG, The PRISMA Group (2009). Preferred Reporting Items for Systematic Reviews and Meta-Analyses: The PRISMA Statement. PLoS Med 6(7): e1000097. doi:10.1371/journal.pmed1000097

For more information, visit: **www.prisma-statement.org**.
