# Supplementary material for: Women’s and girls’ experiences of menstruation in low- and middle-income countries: A systematic review and qualitative metasynthesis
Source: PLoS Med. 2019 May 16;16(5):e1002803. doi: 10.1371/journal.pmed.1002803 (PMC6521998; doi:10.1371/journal.pmed.1002803)
Supplement: S1 Text — (PDF) [file pmed.1002803.s002.pdf]

Supplementary Materials. S1 Text.

Grey literature searching: list of organisation websites

Organisation websites and repositories searched for grey literature:

- CARE
- Days for Girls
- Femme International
- International Rescue Committee (IRC)
- International Federation of Red Cross and Red Crescent Societies (IFRC)
- Irise International
- Menstrual Health Hub ("resources" library)
- Menstrual Hygiene Day ("resources" library)
- Oxfam
- PATH
- Population Services International (PSI)
- Plan International
- Save the Children
- SNV
- Sustainable Sanitation Alliance (SuSanA) ("resources" library and uploads)
- UNICEF
- UNFPA
- UN Women
- USAID
- Water Aid
- WASHUnited
- World Vision
- WSSCC
- WoMena
- ZanaAfrica
