# Supplementary material for: Women’s and girls’ experiences of menstruation in low- and middle-income countries: A systematic review and qualitative metasynthesis
Source: PLoS Med. 2019 May 16;16(5):e1002803. doi: 10.1371/journal.pmed.1002803 (PMC6521998; doi:10.1371/journal.pmed.1002803)
Supplement: S1 Table — (PDF) [file pmed.1002803.s003.pdf]

S1 Table. Quality of included studies

Table 1. Study quality appraisal using the EPPI-Centre tool, with brief notes regarding the rating assigned.

**Colour Key**	**1. Were steps taken to increase rigour in the sampling?**	**2. Were steps taken to increase rigour in the data collected?**	**3. Were steps taken to increase rigour in the analysis of the data?**	**4. Were the findings of the study grounded in/supported by the data?**	**5. Please rate the findings of the study in terms of their breadth and depth**	**6. To what extent does the study privilege the perspectives and experiences of women and girls?**
	Yes, a fairly thorough attempt was made	Yes, a fairly thorough attempt was made	Yes, a fairly thorough attempt was made	Good grounding/support	Good/fair breadth and depth	A lot
	Yes, several steps were taken	Yes, several steps were taken	Yes, several steps were taken	Fair grounding support	Good/fair depth but very little breadth	Somewhat
	Yes, a few steps were taken	Yes, a few steps were taken	Yes, a few steps were taken		Good/fair breadth but very little depth	A little
	No, not at all/not stated/can't tell	No, not at all/not stated/can't tell	No, not at all/not stated/can't tell	Limited grounding/support	Limited breadth or depth	Not at all
	**Overall trustworthiness, consider questions 1 to 4**				**Overall usefulness/relevance, consider the <u>review question</u> and questions 5 and 6**	
	High				High	
	Medium				Medium	
	Low				Low	

**Study ID**	**1. Rigor in sampling**	**2. Rigor in data collection**	**3. Rigor in analysis**	**4. Findings supported by the data**	**5. Breadth and depth of findings**	**6. Privileges perspectives of women and girls**
Adegbayi 2017	Self-selection through fliers at university campus.	Written narratives offer privacy. Limited probing. Topic guide provided.	Strong description of analytic process.	Quotations, no identifiers. Themes supported although some linked to past research more so than primary data.	Some depth and breadth.	Mix of iterative and deductive approach with reliance on past literature rather than primary quotations for some themes.
	Trustworthiness: Medium				Relevance: Medium	
Al Omari 2016	Snowball sampling, many refused. Unclear participant representation.	Written journal approach provided privacy and repeat observations. Guiding questions reported.	Two coders. Limited analytic information provided. Differing approaches reported in abstract and methods.	Rich support of themes with quotations and participant numbers.	Good depth of findings, limited breadth.	Diary approach was girl-centric. Focus on girls' narratives in analysis.
	Trustworthiness: Medium				Relevance: High	
Al-Sabir 1998	Random sampling of regions, followed by purposive sampling of women and adolescent girls.	Mix of IDIs and FGDs, Topic guide not reported, although development process stated. Results suggest many structured questions.	No stated analytic framework. Data presented descriptively with little analysis apparent.	Theme generation and description unclear. Domains of menstrual experience well descried by extensive quotes. No participant numbers.	Study describes broad set of behaviours and factors influencing experience. Depth provided through quotations but limited analysis or synthesis into themes.	Perspectives clear in case studies, however analysis limited.
	Trustworthiness: Medium				Relevance: Medium	
Al-Shurbji 2017	Sample identified by community leaders, process unclear. Participants selected from each camp block, but otherwise unclear characteristics.	Data collection tools informed by past research. IDIs until saturation. Full topic guide reported, structured questions.	Stated use of grounded theory and feminist perspective, but no description of analytic process. No presentation of themes or theory. Saturation noted but not supported	Unclear themes presented. Supportive quotations provided, but numerous strong statements without supporting quotes.	No synthesis into themes. Fair breadth of issues presented but limited depth.	Attentive to religious context however more focus on author interpretation than participants. Unclear involvement of participants in process.
	Trustworthiness: Low				Relevance: Medium	
Amatya 2018	Convenience sample of volunteers from one school.	Single FGD. Data collection tool designed to triangulate quantitative survey. Semi-	Stated phenomenological and thematic approach. Brief description of analytic process.	Findings provided depth to quantitative methods. No themes, responses presented according to answers to topic questions.	Single FGD to inform quantitative interpretation. Little breath or depth in analysis.	Unclear involvement of women and girls. Author focus on participant perspective.

		structured questions explored research questions.	No stated saturation in small sample.	Supporting quotations provided, no participant numbers.		
	Trustworthiness: Low				Relevance: Medium	
Behera 2015	Convenience sample from one school. Specific sample age group. Mix of pre and post menarche and day and boarding students sought.	No ethical approval, informed consent noted. .FGDs, unstructured questions provided but elicited limited depth.	Limited discussion of analytic framework. Themes showed little deviation from topic guide. Attention to divergent cases.	No participant numbers provided. Supportive quotations for themes. Core assertions often supported by multiple quotations.	Broad coverage of experience, but limited depth.	FGD only, fair attempt to privilege girls' perspectives.
	Trustworthiness: Medium				Relevance: Medium	
Bilani 2015	Theoretical sampling described, though specific recruitment methods unclear.	Repeat interviews conducted with several respondents. Respondent checking of interview transcripts.	Multiple analysts read and reread transcripts; respondent validation of analysis; auditable and transparent analysis trail.	Quotes used and tabulated to clarify themes.	Findings generally corresponded to categories rather than specific themes in the data.	Analysis focuses on women's service needs and perspectives, but several details of sampling missing.
	Trustworthiness: Medium				Relevance: Medium	
Boosey 2014	Six schools, purposive selection of girls. FGD participants selected by teachers – eldest and most willing.	FGD (mixed-methods study). Participatory methods stated but none described.	Coding discussed by two researchers. Very limited analysis presented.	No supporting quotations. Limited description of themes.	Very limited breadth or depth reported.	Participatory focus stated but unclear. Emphasis on girls recommendations for solutions to improve menstrual experience.
	Trustworthiness: Low				Relevance: Low	
Budhathoki 2018	Selection of most affected areas and participants. Purposive, though small, sample.	Mixed methods study with limited time (10-15 mins, 5 participants) for qualitative collection. Community engagement process.	Clear description of analytic process. Thematic saturation noted, however very limited sample present to achieve this.	Quotations provided, with participant numbers which support identified themes.	Very limited. Small quantity of interviews of short duration. Mixed-methods study with more attention to quantitative findings.	Engagement with community. Limited qualitative component, but attention to women's perspectives in what was done.
	Trustworthiness: High				Relevance: Medium	
Caruso 2013	Eight schools in two communities and mix of in and out of school girls. Unstated participant selection.	Combination of IDIs and FGDs. Guiding socioecological framework but limited information on topic guide.	No stated analytic approach. Some attention to divergent cases. Themes reflect research questions, no reflexivity noted. Unclear if any iterative coding.	Quotations provided with participant or FGD numbers. Additional longer excerpts from interviews provided.	Socioecological framework approach captured factors at many levels, demonstrating breadth but restricted analytic depth.	Combination of IDIs and FGDs. Focus on girls' suggested improvements, however unclear theme development.
	Trustworthiness: Medium				Relevance: High	
Caruso 2017	Purposive sampling across life course in multiple communities.	Free-list interviews and FGDs. Development of FGD tools based on free list interviews.	Clear description of analytic process. Some attention to divergent cases.	No participant numbers provided, FGD or free-list interview noted. Extensive quotations support themes.	Combination of breadth of sanitation-related challenges and depth.	Combination of methods to take participant centred approach.
	Trustworthiness: High				Relevance: Medium: research question regards sanitation	
Castaneda 1996	Ethnography. Demographic map drawn to assist diverse sample selection. Participatory observation over 2-month period.	Multiple methodologies including interview and observation. Unclear topic guidance for interviews.	Limited description of analytic process beyond stating an ethnographic approach. Reflections on researcher role. Unclear acknowledgement of divergent cases.	Limited quotations, reliance on researcher report. Focus on key terms used in local language.	In depth analysis of meanings of fertility and menarche.	Focus on key informants, midwives, but combined with observation of interactions with women and girls.
	Trustworthiness: Medium				Relevance: Low: limited attention to experience of menstruation.	
Chebii 2018	Selection of oldest-running school. Teacher selected most appropriate class and all girls invited.	Multiple FGDs with the same girls, followed by IDIs to discuss private challenges. Topic guide provided with open ended questions.	Clear description of analytic process. Single analyst, some reflexivity noted. Unclear saturation and presentation of themes identified.	Thematic structure not clearly presented. Multiple supportive quotations provided including participant identifiers.	Significant depth and breadth provided.	Multiple data collection strategies and repeated meetings. Girls' perspective sought and privileged.
	Trustworthiness: High				Relevance: High	
Chinyama 2019	Purposive selection of rural schools in two districts in consultation with Education board. Teacher identification of participants according to eligibility only.	FGDs and IDIs. Topic guides provided. Semi-structured, broad range of topics to elicit narrative and answer research questions. Triangulation with key informants.	Some description of analytic process. Unclear saturation in Methods, Discussion states did not reach saturation. Reflexivity unclear.	Multiple supporting quotations provided with some participant identifiers. Clear description of themes.	Breadth provided with some depth.	Multiple data collection strategies. Girls' perspectives sought and privileged.
	Trustworthiness: High				Relevance: High	

Chothe 2014	Unclear selection of schools. Very high refusal rates.	Data collection method well matched to research question. Limited design (students asking questions).	Unclear analytic approach. Iterative thematic approach apparent and well suited to the question.	Themes identified well supported by example questions. No participant numbers provided.	Broad findings. Very limited depth in analysis. Narrow picture provided by girls' questions.	Single activity: focus on girls' generated question. No steps taken to understand knowledge of girls who did not ask a question.
	Trustworthiness: Medium				Relevance: Low	
Crawford 2014	Convenience sample of university and NGO office women. English fluency required.	Unclear topic guide. Mix of interviews and FGDs.	Clear description of analytic process.	Participant identifiers provided. Themes well supported with quotations.	Good depth and breadth for research question. However, narrow for purpose of this review.	Combination of interviews and focus groups. Inductive coding approach.
	Trustworthiness: Medium				Relevance: Medium	
Crichton 2013	Single informal settlement. Purposive sampling with teachers and community members to identify participants.	Combination of IDIs and FGDs. Some information on topic guide presented.	Clear use of inductive and deductive approaches. Unclear saturation.	Themes well supported by quotes, including participant or FGD number.	Depth and breadth presented.	Combination of IDIs and FGDs. Consultation with key informants. Mix of inductive and deductive approach with past literature.
	Trustworthiness: High				Relevance: High	
da Silva Bretas 2011	Purposive sample (unclear characteristics) of students participating in sexuality education outreach activity.	Single FGD repeated for 3 sessions. Single prompting question on experience of menstruation, unclear prompts.	Clear report of analytic position/theory, some description of analytic process. Unclear saturation, reflexivity.	Quotations for broad themes, but not interpretation provided.	Some breadth and depth provided. Depth driven by review of past literature, unclear participant focus.	Unclear. Integration of results presentation with past literature with little reflexivity on interpretation.
	Trustworthiness: Medium				Relevance: Low	
Daniels 2016	Purposive sampling of areas then random sampling of participants, identified by teachers.	Structured IDIs and FGDs, little room for variation. Detailed topic guide provided. Expert consultation and literature to generate items.	Stated grounded theory approach in analysis, but not in data collection methods. Mix of deductive and inductive coding.	Extensive quotes provided, some participant identification.	Good depth and breadth of findings. Findings across knowledge, practices, impacts, support structure, management.	Mix of inductive and deductive coding. Use of FGDs and IDIs. Triangulation with key informants but focussed on girls' experiences.
	Trustworthiness: High				Relevance: High	
Devnarain 2011	Purposive selection of a single school. Unclear participant selection. 10 students.	Participatory activity to map water activities. Used to generate questions for FGDs. Limited information on FGD facilitation.	Limited information on analytic approach. Mix of inductive and deductive coding. No notes of saturation or reflexivity.	Moderate support through quotations to support key assertions. Participant identifiers reported.	Some breadth and depth of findings.	Use of participatory activity in combination with FGD to focus on girls' experiences.
	Trustworthiness: Medium				Relevance: Low to review question	
Dhingra 2009	Snowball sampling of families, random sampling of girls (unclear method)	IDIs and small group (2-3 interviews), unclear mix of structured questions – broad topic provided.	'Content analysis' reported. No description of analytic process and unclear from results presentation.	Presentation focused on quantitative descriptors. Few quotations provided with no participant identification.	Very little breadth or depth. Little attention to qualitative analysis, largely quantified.	Unclear. Interviews with broad topic guide. Results do not privilege girls' perspectives.
	Trustworthiness: Low				Low:	
Do Amaral 2011	Snowball sampling. Unclear objectives for diversity of participants, most participants connected to university.	FGDs. Divided by age groups. Pilot tested and overview of questions provided.	Description of process. Stated framework. Some missing details of analysis and saturation.	Rich quotations provided, context from past literature. No participant identifiers	Good depth, some breadth.	Mix of participant focus and past theory and literature.
	Trustworthiness: Medium				Relevance: Medium	
Dolan 2014	Range of regions included. Unclear participant identification and recruitment.	FGD and IDI content directed by consultation with stakeholders. No description of topic guide.	Analytic approach not reported. Narrative description of findings. No identified themes.	Unclear themes, however narrative was supported by quotations, no participant identifiers.	Mixed-methods approach but narrative treatment of findings provided some depth and breadth.	Key informant FGDs used to inform questions, followed by emphasis on girls' reports.
	Trustworthiness: Low				Relevance: Medium	
Ellis 2016	Data collection in 13 schools in 3 regions. Urban and rural settings. Purposive participant selection.	FGDs with some information regarding topic guide reported. Topic guide underwent extensive development process.	Limited report of analytic approach. Mix of deductive and inductive coding, with follow up paper appearing to have only deductive approach.	Some supporting quotations, more provided in source report than peer-review paper. Some participant identifiers.	Limited depth. Comprehensive coverage of WASH challenges and girls' experiences.	Deductive approach, with experiences categorized according to framework. FGDs only. Part of longer process of investigation.
	Trustworthiness: Medium				Relevance: High	
Garg 2001	Range of ages, all participants symptomatic of RTI. Unclear participant recruitment.	Some information provided on topic guide. IDIs and FGDs.	Analytic process not reported.	Supporting quotations provided, no participant identifiers.	Breadth and some depth of findings.	Focus on women's experience. Consultation with key informants and health services.
	Trustworthiness: Medium				Relevance: High	

Girod 2017	Purposive selection of six schools on range of demographic and environmental factors. Participants selected by teachers.	Participatory FGD activities, anonymous question session.	Well described analytic process.	Supporting quotations for some points, although some assertions made without supporting evidence. Participant identifiers provided.	Good breath and some depth of analysis.	Participatory activities to encourage participation. Mixed with facility observation and key informants.
		Trustworthiness: High			Relevance: High	
Guerry 2013	Data collection in two schools. Only seven interviews. Mixed methods with greater emphasis on quantitative analysis.	Interview topic guide provided. Private locations. IDIs only and limited sample.	Described analytic process and discussed reflexivity. Unclear saturation.	Supporting quotations provided with participant identifiers. Limited description of themes.	Limited attention to depth or breadth of qualitative analysis and more emphasis on quantitative results.	Limited number of structured interviews. Unclear prioritisation of girls' experience.
		Trustworthiness: Medium			Relevance: Medium	
Hosseini 2018	Unclear recruitment of sample of university students. No further sample description provided.	Limited information provided on topic guide – menarche and menstruation experience. Informed by literature review.	Analysis clearly described. Validation with senior researchers and participants (although method unclear). Saturation unclear.	Themes supported by quotations (no participant identifiers). Restricted author description of themes.	Some depth and breadth of results presented.	Author notes validation of themes with participants but unclear method. IDIs, women's reports clear.
		Trustworthiness: Medium			Relevance: Medium	
Ismail 2016	University students only. Recruited through lectures. Self-selected. .	Limited information regarding topic guide. Questions derived from literature. Three FGDs.	Discourse analysis stated, little description of analytic process, reflexivity or saturation.	Themes well supported by quotations with participant identifiers. Findings integrated with past literature.	Depth of discourses described as per research question, and good breadth according to research question.	FGDs only and integration with literature for both question creation and interpretation of results. Women's reports clear in presentation of quotations and themes.
		Trustworthiness: Low			Relevance: Medium	
IWDA 2017	Purposive sampling via partner organization networks. Largely convenience sample.	FGDs and IDI with girls and women. Topic guide informed by past research and theory. Participatory activities including body mapping, community mapping, 'ideal' latrine.	Inductive coding framework during data collection and followed up afterwards. Findings validated by local research team. Discussion of saturation.	Report notes saturation was not reached. Supporting quotations provided with some identification. High rating for peer-reviewed publication on restrictive practices.	Good breadth, some depth.	Use of participatory activities and some interviews. Local researcher engagement in a analytic feedback. Mix of focus on women's reports and field observation.
		Trustworthiness: Medium			Relevance: Medium	
Jewitt 2014	Nine schools with range of characteristics. Rural and urban areas. Unclear participant sampling.	Multiple participatory activities. Combination of IDIs and FGDs.	Unstated analytic strategy, reflexivity or saturation.	Integration with past literature. Supportive quotations provided.	Depth and breadth described.	Participatory activities prioritised girls' perspectives. Triangulation with key informants.
		Trustworthiness: Medium			Relevance: High	
Kansal 2016	Multi-stage sampling for quantitative survey. FGD participants randomly sampled from survey, balanced characteristics.	FGDs. No topic guide reported, very little information on questions or objectives for FGDs.	No clear description of analytic process. No presentation of themes in results.	No presentation of themes. Largely quantified qualitative responses. Few supporting quotations without identifiers.	Very little depth or breadth presented.	FGDs only. Largely quantitative study. Qualitative data to illustrate quantitative findings rather than participant perspectives.
		Trustworthiness: Low			Relevance: Low	
Krishnan 2016	Unclear participant recruitment for FGDs. IDIs selected from women-headed households purposively and followed up.	Unstructured interviews, FGDs with participatory learning and action tools. Some information on topic guide, no description of participatory activities.	Thematic analysis, but little information on process described. No attention to divergent cases.	No supporting quotations provided. Some description of themes and insights with links to past literature. Unclear analytic strategy and use of primary data to derive themes.	Limited breadth and depth of presented themes. Insights split between description of sites and thematic analysis of content.	No use of quotations, methodology describes extensive consultation. Poor translation to findings.
		Trustworthiness: Low			Relevance: Low	
Kyomugisha 1999	Participant selection not reported.	Participatory listing exercise. Use of IDIs, FGDs with girls, parents and teachers. Unclear composition.	Unstated analytic approach. Two stage process of listing and interviews. Unclear derivation of themes.	Supporting quotations provided, no participant identifiers. No derivation of themes.	Restricted research question. No themes presented. Some depth on single issue.	Mix of girls' and teachers/parents experience. Unclear involvement of girls in study process.
		Trustworthiness: Low			Relevance: Low	
Lahme 2016	Three schools. Schools selected for varied characteristics. Purposive sampling of participants.	FGDs. Description of topic guidance. Concurrent analysis and data collection. Findings validated with participants.	Clear description of analytic process and participant validation. Notes on reflexivity.	Supporting quotations provided, no participant identifiers. Consistency between themes and supporting quotations.	Broad and deep themes presented consistent with research question.	Participants provided feedback on preliminary analysis. Prioritisation of women's perspective.
		Trustworthiness: High			Relevance: High	

Long 2013	Ten schools and surrounding communities. Schools purposively sampled. Unclear participant sampling.	FGDs and IDIs. Some information on topic guide. Participatory board game and small group activities to promote comfort.	Unclear analytic process. Deductive approach from existing framework. Stakeholders provided feedback on study results. FGDs with participants for validation.	Supporting quotations with some participant identification. Supporting photos and case studies.	Extensive breadth and so depth into identified findings.	Unclear deductive vs inductive coding. Mix of girls' and informant perspectives.
			Trustworthiness: Medium			Relevance: High
Mason 2013	Six schools, all eligible girls per school participated.	Some description of topic guide topics. FGDs.	Thorough description of analytic process. Consideration of saturation.	Themes supported by quotations and participant numbers. Consistency between themes and quotations.	Breadth and depth of findings.	Unclear development of tools. Strong reliance on girls' experiences, triangulation key informants.
			Trustworthiness: High			Relevance: High
McMahon 2011	Six rural schools, unclear sampling strategy. Teachers and school staff selected participants.	FGDs. Open ended interview questions. Very little information on topic guide provided.	Thorough description of analytic process. Notes saturation and two-phase design. Reflection on researcher role.	Supporting quotations provided, no participant identification.	Coverage of domains, and depth of themes presented.	Unclear development of tools. Strong reliance on girls' experiences, triangulation with teacher perspectives.
			Trustworthiness: Medium			Relevance: High
Miiro 2018	Four purposively selected schools. Random sample of girls from each grade level.	Some information on topic guide. Use of participatory methods noted. FGDs followed up with IDIs.	Thorough description of analytic process. Reflexivity and saturation absent. Steps taken to ensure discussion and reliability across coders.	Supportive quotations provided with focus group numbers. Presentation of qualitative themes integrated with quantitative results.	Some breadth and depth. Mixed-methods approach, and restricted space for qualitative findings.	Mix of FGDs and IDIs, privileging of girls' experiences, triangulation with key informants.
			Trustworthiness: Medium			Relevance: High
Morowatisharifabad 2018	Unclear school selection (both private and public), unclear participant selection	Limited information on topic guide. Interviews only. Both girls and parents interviewed (unclear if connected). Stated follow ups with participants.	Clear description of analytic process, multiple coders. Data collection continued until saturation.	Supportive quotations provided. Themes checked with participants and experts. Participant identifiers reported.	Specific research question; breadth provided for this question. Some depth.	Validation of findings with research participants. Multiple perspectives from girls and parents and key informants. Participants checked themes.
			Trustworthiness: Medium			Relevance: High
Morrison 2016	Twelve schools in mix of areas. Unclear selection of participants.	Pair interviews, FGDs used game, storytelling and body mapping to enhance communication. Menstrual products distributed for discussion.	Thorough description of analytic process, identification of deviant cases. Multiple coders.	Supporting quotations provided with participant numbers.	Breadth and depth of findings presented.	Mix of group interviews and FGDs. Participatory activities. Triangulation with key informants but privileging of girls' experiences.
			Trustworthiness: High			Relevance: High
Mumtaz 2016	Implementing partner selected sites. One school. Out of school girls identified by social worker. Unclear recruitment of in school girls.	Participatory activities in FGDs. Multiple sessions with girls. Menstrual stories, brainstorming WASH facilities.	Some description of analytic process. Limited reliability checks, no reflection on reflexivity, deviant cases.	Quotations provided with participant identifiers.	Good breadth of coverage and some depth.	Multiple participatory activities over many days. Triangulation with observation and key informants. Girls' experience privileged.
			Trustworthiness: Medium			Relevance: High
Naeem 2015	Six schools selected in two provinces. School going girls. Unclear participant selection.	FGDs with girls. No information on topic guide or process.	No stated/clear analytic process.	No supporting quotations provided. Only observational information and key informant reports.	Breadth of issues, but no depth and no supporting quotations.	Unclear.
			Trustworthiness: Low			Relevance: Low
Nanda 2016	Selection of urban and rural schools, otherwise unclear. Teachers and school officials identified adolescents for participation.	FGDs. No presentation of topic guide some description on topics discussed.	No stated analytic process.	Illustrative quotes presented. No participant numbers.	Reasonable breadth and some depth.	Unclear FGD activities. Emphasis on girls' voices. Triangulation with key informants.
			Trustworthiness: Medium			Relevance: High
Narayan 2001	All schools selected in urban and rural areas. Schoolgirls 12 – 17 and older women. Unclear participant selection.	IDIs and FGD. No presentation of topic guide. Topics of interest briefly discussed.	No stated analytic process.	No illustrative quotes, no participant identification. No themes presented only narrative description of initiation process and quantiative data.	Limited breadth and depth. No supporting quotations.	Participatory activities included during qualitative interviewing (e.g. body mapping). Quantitative survey tool developed based on qualitative findings.
			Trustworthiness: Low			Relevance: Low

Nechitilo 2016	Selection of schools representing language groups, with poorer WASH facilities. Self-selected volunteer participants recruited	FGDs and IDIs. Very broad topic guide provided. Validation meetings with sub-set of participants.	Analytic process reported. Deductive use of existing codes, some inductive themes.	Illustrative quotations presented with participant identification and stories.	Extensive breadth of findings, some analytic depth.	Deductive coding based on work in other locations. Validation meetings with participants. Triangulation with key informants. Multiple data collection strategies.
			Trustworthiness: Medium			Relevance: High
Padmanadbhanu-nni 2017	Undergraduate or postgraduate students. Study advertised on student notice boards and mailing lists – self-selected participants.	5 IDIs, 3 FGDs. No discussion of saturation. Example questions provided indicative of approach.	Description of analytic process. Notes checking for consistency and divergent cases. Independent audit of analytic process.	Supporting/illustrative quotations provided. No participant identifiers of IDI or FGD.	Reasonable depth and breadth.	Mix of IDIs and FGDs. Focus on women's experiences. Unclear process of question development.
			Trustworthiness: Medium			Relevance: Medium
Parker 2014	Girls selected by teachers. Women asked to volunteer (unclear recruitment). Camps selected to vary on size, age and geography.	FGDs with women and girls. Noted emphasis on views of the community and action research, however no description of topic guide or question development.	Reflexivity throughout and stated efforts to reduce bias by looking for divergent cases. However, analytic process not described.	Supporting quotations provided although not for all key points, some participant identification.	Broad findings, some depth for included themes.	Stated participatory approach, unclear involvement of population in reflecting on findings. Focus on women and girls' triangulated with key informants.
			Trustworthiness: Medium			Relevance: High
Pillitteri 2011	Seven schools, mix of characteristics. Unclear participant selection.	Participatory group workshops. Toilet drawing, list of priorities, writing first menstrual experience, anonymous questions, puberty curriculum.	No stated analytic approach. Unclear deductive or inductive process.	Supporting quotations provided with identifiers. Unclear mix of findings from girls compared to past research, key informants and researcher observation.	Broad finding, little reciprocal translation from interviews in depth.	Unclear themes from girls compared to past research.
			Trustworthiness: Medium			Relevance: Medium
Person 2014	Schools in three regions, selected for sufficient size and age of students. Unclear participant selection or characteristics.	Mix of FGDs and IDIs, key informant interviews. Questions piloted with multiple respondents. Broad topic guide provided.	No stated analytic approach. No report of analytic process. NO reflexivity, saturation.	Limited themes presented. Unclear attention to divergence. Supporting quotations provided (no participant identifiers)	Breadth of findings but very little analytic depth. Categorical identification of 'what is needed'.	Tool piloted. Triangulation between girls, parents, key informants, community actors. Some prioritisation of girls' perspectives.
			Trustworthiness: Low			Relevance: Medium
Rheinlander 2018	Single community, two schools. Teachers selected girls for participation (who were willing to talk openly about the subject). Selected girls identified 3-4 friends to join discussion.	Emphasis on interaction with community and triangulation. FGDs with girls. Thematic guide reported including use of vignettes. Transect walks.	Clear description of analytic process.	Supporting quotations provided. No participant identification. Multiple responses within FGDs displayed for strong illustration of themes.	Breadth and depth of themes provided.	Unclear process of question development, guided largely by past research. Prioritization of girls' perspectives.
			Trustworthiness: High			Relevance: High
Suduvac 2017	Single focus group in single school. Participants with dignity kits selected, poor concordance with objectives to understand experience.	Mix with desk review and quantitative survey. Limited information provided.	No stated analytic process.	Supportive quotations provided, no participant identifiers.	Breadth, very limited depth.	Single focus group only, unclear question derivation.
			Trustworthiness: Low			Relevance: Low
Schmitt 2017	Two different humanitarian populations. Purposive sampling for diverse age groups, ethnicity and living situation.	FGDs with girls and women, and participatory mapping activities. Some information on topic guide. Map of communities and identify locations for menstrual activities.	Description of analytic process provided. Unclear reflexivity or saturation. Unclear identification of divergent cases.	Some supportive quotations provided. No participant identifiers. (more quotations from KIIs than women and girls, no recording of FGDs to support privacy)	Good mix of breadth and depth.	Mix of focus between women and girls experience and key informants.
			Trustworthiness: High			Relevance: High
Scorgie 2016	Sites selected based on relationships with stakeholder in the region. Participants selected through social network mapping from larger study.	Photovoice, participatory workshops (FGD including body mapping) and IDIs.	Strong description of analytic process.	Supporting quotations provided with participant identifiers and descriptions.	Strong mix of breadth and depth.	Participatory and photo-voice approach privileges women's lived experiences.
			Trustworthiness: High			Relevance: High

Secor-Turner 2016	Participants recruited from single community and recruited through teachers and community members.	IDIs. Topic guide provided. Girls in schools invited to submit written questions.	Adequate description of analytic process. Engagement with community nurse in study design.	Supporting quotations provided. No participant identifiers.	Limited word length. Some breadth and depth provided.	Engagement with community and focus on girls' experiences.
	Trustworthiness: Medium				Relevance: Medium	
Singh 2006	Four FGDs. Unclear participant recruitment.	Multiple key informant interviews used to inform interview schedule. No description of topic guide provided.	No description of analytic process.	Thematic groupings supported by illustrative quotations. No participant numbers.	Some breadth, limited depth.	Development of questions through engagement with key informants. Reporting privileges women.
	Trustworthiness: Low				Relevance: Medium	
Sommer 2009	Two districts (rural and urban). School and vocation training centre in each district. Girls selected by teachers informed to support diverse sample.	Participatory activities. Menstrual narratives (written), puberty questions, designing 'growing up' curriculum. Multiple meetings with students.	Description of analytic process. Single coder only. Unclear saturation, reflexivity, divergent cases.	Supporting excerpts provided with participant identifiers.	Extensive breadth and depth facilitated by three publications.	Unclear derivation of activities and questions, privileging of girls' experiences and perspectives throughout.
	Trustworthiness: High				Relevance: High	
Sommer 2015	Four countries selected through engaged partners. Mix of rural and urban sites in each country.	Participatory activities (menstrual stories, brainstorming how school environment could be improved, 'perfect toilet'). Multiple meetings over 4 weeks.	Description of analytic process. Feedback from experts. Ongoing analysis. Unclear divergent cases or reflexivity.	Supporting quotations provided, some participant identification. Some findings lack supportive quotations. .	Breadth and some depth provided across two publications.	Activities based on past research. Multiple meetings with girls and activities. Input from local experts noted in data analysis.
	Trustworthiness: High				Relevance: High	
Sosa-Sanchez 2014	Theoretical sampling in four different neighbourhoods from one state.	IDI supported by topic guide provided, informed consent and ongoing analysis.	Strong description of analytic process with attention to contextual contingencies and deviant cases.	Extensive quotations provided.	Good depth and breadth of findings offer theoretical insights and engagement with diverse viewpoints.	Women's perspectives clear and accounted for in reflexivity.
	Trustworthiness: High				Relevance: High	
Timaru 2015	5 countries, but different studies undertaken. Report & publication provide 'summary of findings'. Unclear participant selection – limited methodological reporting for any country.	FGDs with girls in some locations. Very limited methodological reporting. Unclear FGD questions and process.	No stated analytic strategy for reporting across findings or for individual studies reported on.	Some questions provided. Unclear theme derivation. Quotes largely provided as illustrative to support quantitative findings.	Not adequately reported.	Unclear. Reporting largely dependent on quantitative data with little integration of qualitative findings.
	Trustworthiness: Low				Relevance: Low	
Tegegne 2014	Participants selected from different school clubs. Drop outs purposively selected. All participants recruited through female teachers. Unclear school selection.	IDIs (n=5) and 4 FGDs. Some information regarding topic guide provided.	Clear description of analytic process. Saturation noted. Discussion between investigators. Unclear reflexivity or divergent cases.	Supporting quotations provided, with some identification. Mixed methods reporting.	Mixed methods reporting, limited space available for in-depth qualitative analysis. Breadth but lacking some depth.	Unclear development of topic guide or input from women and girls. Some privileging of girls' experiences, mixed with pre-formed survey questions from quantitative work.
	Trustworthiness: Medium				Relevance: Medium	
Thakur 2014	Unclear participant selection alongside random sample design for survey.	No topic guide for FGDs provided. Pre-testing of survey noted, but unclear for FGDs.	No description of analytic process beyond 'content analysis' no description of themes.	Few quotations to illustrate quantitative findings.	Some breadth but largely qualitative. Very little depth.	Qualitative used to illustrate quantitative rather than for in depth analysis. Little privileging of participant perspectives.
	Trustworthiness: Low				Relevance: Low	
Trinies 2015	Eight urban and rural schools in two regions. Schools selected purposively. Study staff selected participants.	IDIs with girls. Very short description of topic guide.	Brief description of analytic process. Unclear discussion between researchers, saturation.	Supporting quotations provided, with some participant identification.	Adequate depth and some breadth provided.	Some privileging of girls' experience, use of deductive coding from past work.
	Trustworthiness: Medium				Relevance: Medium	
Ullrich 1992	Single village ethnography. Unclear sampling.	Unclear data collection. Community followed by researcher for many years.	Not reported.	Unclear. Limited supporting quotations.	Depth, and some breadth.	Unclear.
	Trustworthiness: Low				Relevance: Low (research question limited relevance for review)	

Umeora 2008	12 rural communities selected by project director blinded to objectives. Women encountered by chance. IDI participants recruited from study communities.	32 open ended survey questions. IDIs with 12 women >50 years. Unclear topic guide.	No description of qualitative analysis. No themes presented.	Some supporting quotations provided although no participant identifiers. No themes presented.	Limited breadth or depth.	Unclear question development, analytic process or reporting.
	Trustworthiness: Low				Relevance: Low	
UN Women 2017	Two regions selected for qualitative component. Purposive snowball sampling to access participants who met requirements.	FGDs with women and girls (n=5). Unclear topic guide.	No description of analytic process.	Some supporting quotations provided. Most illustrative of quantitative data rather than any iterative development. Some identification of challenges through qualitative approach.	Lots of breadth but driven by quantitative component, unclear qualitative component.	Unclear. Some use of qualitative data but unclear use of iterative coding, largely illustrative.
	Trustworthiness: Low				Relevance: Medium	
Wall 2016	Randomly selected households in 10 subdistricts (split of urban and rural).	Semi structured survey approach with open ended questions. Open-ended questions reported.	No analytic process reported. Largely quantification of open text responses. No themes.	Very limited quotation provided. Largely illustrative of quantitative approach.	Breadth, but very little depth provided.	No iterative themes. Semi-structured approach sought respondent-driven information but limited depth for qualitative study.
	Trustworthiness: Low				Relevance: Low	
Wall 2018	Region selected alongside above study household survey. Selection of participants for qualitative study not reported.	No topic guides reported. Combination of IDIs and FGDs. Range of participants including both menstruating and not, and key informants.	No details provided on analytic process beyond 'synthesis to themes'. No reflexivity, saturation not noted. Unclear participation of authorship team.	Supporting quotations provided with some participant identification. No reported validation with participants.	Breadth of findings presented with contextual depth.	Unclear role of participant perspective in narrative summary produced. Mix of women's perspective with elders and males.
	Trustworthiness: Medium				Relevance: Medium	
WaterAid 2009	Four districts selected purposively. One school per district. 'Articulate and willing' girls selected by teachers. Girls with 'noteworthy experience of school absence or other problems' selected for IDI.	FGDs, 1 per school. IDIs with one or two girls in each school. No stated topic guide.	Unclear analytic process.	Quotations sometimes illustrative of quantitative findings. Some case studies presented of worst experiences. Some emergent themes from qualitative work. No participant identifiers.	Reasonable breadth, some depth.	Unclear identification of themes. Mixed-methods approach but insufficient information on qualitative process.
	Trustworthiness: Medium				Relevance: Medium	
WWSSCC 2014	Diverse region. 'menstrual hygiene management lab' set up. Convenience sample, unclear recruitment. Large sample.	FGDs. Vague topic guide. Stated observations of infrastructure but unclear methodology.	No stated analytic process.	Some supporting quotations provided, not for all assertions. No participant identification.	Broad range of topics covered, but very limited depth of qualitative analysis.	Mixed methods approach but unclear focus on women's reports compared to quantitative survey, past research and policy.
	Trustworthiness: Low				Relevance: Medium	
**Disorders and pain**						
Aziato 2014	Purposive sampling until saturation. Female university students and high school.	Some limited information provided on structured interview guide. IDIs.	Clear description of analytic process. Validation with participants. Saturation noted.	Supportive quotations illustrate derived themes.	Breadth and depth of findings reported, focused on pain only.	To a high degree. Findings validated with participants, analysis prioritise lived experience.
	Trustworthiness: High				Relevance: Medium	
Hemachandra 2009	Purposive sampling for more highly educated women in 6 geographic areas until saturation. Women with problems selected for follow up interviews.	Some information on individual interview topic guide provided. Unclear topic guide for FGDs.	Clear description of rigorous analytic process.	Findings supported by illustrative quotations with participant identifiers.	Breadth and depth of analysis reported.	Clear prioritisation of women's perspective, triangulation with key informants. FGDs and IDIs used.
	Trustworthiness: High				Relevance: Medium	
Kavitha 2014	Purposive sampling stated but no description of criteria, assumed some report of painful menses.	No description of topic guide.	Some description of analytic process. Unclear saturation. Single coder.	No supportive quotations provided. Only superficial description of themes.	Some breadth, no depth of themes reported.	Unclear use of women's voices in analysis.
	Trustworthiness: Low				Relevance: Low	
Titilayo 2009	Unclear participant selection for qualitative component.	Brief information on interview topics.	Stated Grounded theory, some description of coding process.	Limited qualitative quotations provided. Arguments supported with quotations, no participant identifiers.	Some breadth, very little depth.	Some prioritisation of girls' perspectives. Unclear integration of qualitative with quantitative methods.

	Trustworthiness: Medium				Relevance: Medium	
Walraven 2002	Random sampling of women from each disorder group following cross-sectional survey	Brief information on interview topics provided. Unclear development.	No description of analytic process.	Quotations provided describing disorders but not supporting other statements made in results. Little presentation of analysis.	Little breadth and no depth provided.	Unclear prioritisation of women's perspective. Some explanation of women's disorders in their own voice.
	Trustworthiness: Low				Relevance: Low	
Wong 2011	Recruitment from 11 schools in two districts. Some purposive sampling.	Topic guide described. FGDs grouped by age, ethnicity.	Limited description of analytic process.	Broad themes supported by illustrative quotations with participant identifiers.	Some breadth, little depth of themes.	Analysis privileged girls experience, unclear involvement of women and girls in research process.
	Trustworthiness: Medium				Relevance: Medium	

FGD = Focus Group Discussion. IDI = Individual (In-depth) Interview).
