# Supplementary material for: Women’s and girls’ experiences of menstruation in low- and middle-income countries: A systematic review and qualitative metasynthesis
Source: PLoS Med. 2019 May 16;16(5):e1002803. doi: 10.1371/journal.pmed.1002803 (PMC6521998; doi:10.1371/journal.pmed.1002803)
Supplement: S2 Table — (PDF) [file pmed.1002803.s004.pdf]

S2. Table.

Table 1. Illustrative quotations highlighting the range of menstrual practices undertaken, and for themes of perceptions of menstrual practices and perceptions of menstrual environments

Practice/ Environment	Illustrative quotation	Perception	Illustrative quotation
**Menstrual materials**			
Material used	Almost all of the women interviewed used cloth from old, worn clothes to absorb their menstrual blood, or more rarely, cloth bought from a health clinic…. The cloth, which lasted anywhere from one to six months depending on age at time of first use as a menstrual cloth, was held in place by a waistband (see Figure 2) or, less frequently, by underpants. Some more affluent village women or school girls used disposable sanitary pads with underpants. (Parker 2014)<br><br>"I use old ragged clothes from the children, my own pantaloons, whatever; I try my best to use cotton but if it is not available then in an emergency I use any cloth that is available" (Garg 2001)<br><br>When pads were not available, participants described alternative materials, such as clothing and mattress fibers, to manage their menstruation. (Secor-Turner 2016)<br><br>"In my bag, there is always an old cloth which I use to clean my shoe, I used part of it to make [a] pad" (Caruso 2013)	Acceptability	"If I had an option to choose between sanitary pad and rag cloth (piece of cloth from sari or other cloth)… of course, I would choose the sanitary pad." (WaterAid 2009)<br><br>"We want to use pads when we have to travel, otherwise we want to use cloths when we stay at home" (Morrison 2016)<br><br>"I choose rags because sometimes there is no money to buy pads so these help." (McMahon 2011)<br><br>"Our mothers, grandmothers and even those before them use cloths during their periods and they never had any problems. These (sanitary) pads that 'oyibo' has brought is the cause of many problems women have today as they do not allow (menstrual) blood to come out freely and bring out all the disease". (Umeora 2008)
		Comfort	Girls talked about disposable pads or cloth in terms of the extent to which they could give them 'rashes' and 'itching', which was bad for their health. A rash could occur from having to walk a long way, causing chaffing with cloth, or having to pull off a cloth that had stuck to the vagina and pubic hair. (Morrison 2016)<br><br>"The pads turn over for [the] sticker isn't good. The pads are thin and short, [and there is] too much blood. Accidents happen especially when sleeping and playing." (Daniels 2016)
		Reliability (product)	Girls reported multiple problems with menstrual cloths smelling or falling out at school. (Pilletteri 2011)<br><br>"Sometimes when you are running you feel as if it will fall out and leak." …: "There is nothing to make it stick! At times they fall down when you are walking!" (Parker 2014)
		Reliability (access)	Girls often bought themselves pads in bulk if they travelled into [location] or the town centre. One girl noted: "If it [a supply of sanitary pads] runs out, I use cloth. I bring them [pads] from the city. If I go, I bring myself a lot." Often the city was brought to them via weekend fairs where girls could purchase sanitary pads. (Long 2013)

**Changing Materials**			
Change frequency	Some participants used pads but changed them infrequently, for example, making use of just two or three pads during a whole period lasting 3 or more days. (Crichton 2013)	Acceptability/ comfort	Some women and girls complained that using cloths or infrequent changes of pads irritates their skin and causes rashes. (Crichton 2013)
Environment for changing materials		Cleanliness<br>Privacy<br>Safety	Privacy appeared to be a greater concern for younger girls than adult women, because they often shared the house with parents and siblings and had less control over the physical space within their homes. (Crichton 2013)<br><br>"It is difficult to change in the toilet. Although there are many toilets they are all dirty," (Morrison 2016)<br><br>“Because boys are hard-headed; sometimes they enter intentionally. When we tell them that it’s occupied, they won’t believe us. But when our teacher is around I’m comfortable.” (Ellis 2016)<br><br>Nighttime use of latrines also posed challenges, with many girls and women citing fears of violence, kidnapping or snakes. (Schmitt 2017)
**Storing materials**			
Method and location of material storage	… after use, menstrual cloths were typically washed, hung or lain somewhere private to dry like inside the latrine or under their bed, and stored for re-use in a concealed place (Trinies 2016)	Acceptability	
**Transporting materials**			
If materials are transported	Even when menstruating regularly, very few girls reported bringing materials to school with them in anticipation of their period. Rather, many girls would react to their menses as it arrived, a difficult strategy for menstrual hygiene management because most girls did not bring pads to school, and schools did not provide pads. Girls reported asking friends, teachers or women who lived close to the school for help, or simply going home to manage the onset of menses (Caruso 2013)	Acceptability	When probed on why they did not bring their own materials, some girls cited fear that fellow pupils would see the materials in their bags while others had not thought of bringing replacement supplies or did not have a sack in which to carry them. (Trinies 2015)<br><br>“Some days we bleed heavily, and we need to change clothes at least 2 or 3 times during the school hours. There is no place to change and dispose the cloth – there is question of putting back those used cloth in our pockets. So we just bunk classes when we have to change the clothes.” (WaterAid 2009)
Method of material transport	They carried their menstrual materials to and from school in black plastic bags and claimed not to be teased or bothered by boys when going to the latrines with the bags (Parker 2014)<br><br>Taking absorbent materials home for washing or disposal required girls to inconspicuously bring their school bag to the bathroom with them and place their used menstrual materials inside. Over the course of the school day, girls could accumulate several used menstrual pads or cloths in their school bags. (Long 2013)	Acceptability	“I wrap up the towel and put it in my bag. Then, once I get home, I wash it before disposing it off. I’m afraid that people will see me or that it will smell. I hurry home as quickly as I can.” (WSSCC 2014)

**Clean hands**			
Handwashing before/after menstrual management	Because of lack of water and hand-washing facilities near pit latrines, regular hand-washing after changing MHPs [Menstrual Hygiene Products] tended to be ad hoc, especially when water supply was curtailed without warning. (Scorgie 2016)<br><br>The suggested reasons for the water being available inside the latrine was to enable girls to wash off any signs of blood from their hands… (Sommer 2009)	Acceptability	"They should put water and soap in our toilets because most of the time you find when our tap is locked even when you want to clean yourself up you cannot" (Miiro 2018)<br><br>"We go to the toilet, and then we eat with shit or blood on our hands." (Pillitteri 2011)
Environment for handwashing		Cleanliness<br>Privacy	"Sometimes we go to the sea to wash and rinse and then come to the tap stand to bathe ourselves. First the blood is cleaned in the sea so it is not seen at the tap stand" (IWFD 2017).<br><br>The shame of leaving a latrine or toilet with blood spots from insufficient water to wash it away, or to privately wash blood off their hands, makes toileting in school a stressful event for many girls. (Sommer 2015)
**Clean genitals**			
Method of genital cleaning	Most participants in FGD told that they wash their genitals with water only when they go to toilet. (WaterAid 2009)<br><br>Most bathrooms in the Myanmar camps and informal settlements of Lebanon also lacked a water source inside or convenient to the latrines. This was especially challenging for the Syrians living in informal settlements, who expressed desires for the provision of pre-wet napkins or baby wipes to be included in distributions to help address these hygiene issues. (Schmitt 2107)	Acceptability	Many girls emphasized the importance of showering before school to avoid problems with odors, which contributed to fear shame and distress: "[I manage my MHM at school] by wearing sanitary pad and change it frequently. Before coming to school, [I] need to take shower [to] avoid the smell at others." (Daniels 2016)<br><br>"I had never had a bath while having my periods, as people often say, it might aggravate the bleeding" (Tegegne 2014)
Frequency of genital cleaning during menses	"When blood is coming out you need to be bathing because if you don't bathe you will be smelling, people they will know that you are having period, so you need to be bathing, you do so in the bathroom, bathe nicely and make yourself clean then wear another pad…" (Nanda 2016)<br><br>In each of the three countries, women and girls commonly expressed a preference to bathe or shower each time they change sanitary materials, or at least several times during the day. Often this was explained as being necessary to reduce odour and for comfort. (IWDA 2017)	Acceptability	Sometimes girls had to compete with family members for scarce resources such as soap and water, which could cause conflict, even with mothers. 'Sometimes you want to take a bath three times and your mother quarrel's you that you are misusing her soap.' (P5 School J). 'Your parent will wonder why her daughter is bathing three times which she never does daily, and maybe you didn't fetch water for bathing so you will be forced to use water that was stored in the house and she might say you are misusing her water.' (P6 School D). (Mason 2013)<br><br>"May be when you go bathing there, all of the other people will know that you are in your menstrual period." (McMahon 2011)
Environment for cleaning genitals		Privacy<br>Safety	"There was no soap to take bath and I had to change before the sun came out." (Budhathoki 2018)

			“May be when you go bathing there, all of the other people will know that you are in your menstrual period.” (McMahon 2011)<br><br>There were also no private facilities for body washing. The open-plan showers in boarding schools were unpopular, even when they could be used, though lack of water often prevented this. (Pillitteri 2011)
**Disposal**			
Method and location of material disposal	They discussed throwing pads in ponds, rivers and forested areas: I only use (pads)…If I throw it (outside), they will know it is mine. I will feel bad. If there was a toilet, would have put it there… Here, I throw in the jungle. (Caruso 2017)<br><br>…for the majority of girls, disposal options generally involved burying, burning, or non-specifically throwing their used sanitary materials away from their homes, particularly if a trash receptacle was not an option: “[I] put [used materials] in [a] black plastic bag and burn [it] at the back of my house” “[I] put [used materials] in the black plastic bag and throw away far from home.” (Daniels 2016)	Acceptability to the individual (incl. cultural acceptability, privacy)	“My mother advised me to bury it or throw it in the pit latrine. People say that otherwise there could be witchcraft practised which could impair fertility and close the mouth of uterus… So I bury it” (Garg 2001)<br><br>“When you throw it in the toilet, the next person will know it was you and tell others.” (Crichton 2013)<br><br>Some girls reported difficulties with burying pads as dogs may discover them, flies gather around the waste pit, and the disposed menstrual cloths make a ‘foul smell’ when buried or burnt. (Morrison 2016)
		Reliability	“during [the] raining season, [it is] difficult to bury sanitary pad.” (Daniels 2016)
		Acceptability to others (incl. public health)	“I used an old cloth during my menstrual periods. My neighbor found me a secluded area behind the trees where I was told that other women also changed their pads/cloth. I was horrified at the small pile of sanitary cloth disposed openly just a little distance away.” (Budhathoki 2018)<br><br>Sometimes girls threw their used pads on the floor if they could not wait for a stall with a disposal bin, scaring younger children who would urinate or defecate on the floor to avoid going near the used pad. At one school, the janitor refused to clean the toilets when girls threw their pads on the floor; his frustration caused fear and stress for girls. (Girod 2017)<br><br>menstrual waste discarded in pits and drains of school toilets routinely created problems for toilet functionality and that menstrual waste in surroundings attracted pests. (Rheinlander 2018)
**Washing materials**			
Method of washing stains from outer-garments	Girls described wanting water to wash their hands, wash stains out of their skirts and, in some countries, wanting a place to wash their bodies. (Sommer 2015)		
Amount of water used	They all washed their cloths in soap and water in theory, however many struggled to have enough water or enough, if any, soap. “If I find the soap I can use the soap. If I find the water I can use the water” (Guerry 2013)	Acceptability/ Reliability	“But in school – there is no hot water to soak/wash the cloths” (Sommer 2009)<br><br>I wash the cloths with plain soap and water. I don’t have Dettol (anti-septic solution), so I don’t use it. (Behera 2015)

Vessel for water		Acceptability/ reliability	We also need more basins, the ones we have now are broken and we have to wash everything in them – our rags, our babies. Everything is put into the same basin (Parker 2014)
Soap used	They all washed their cloths in soap and water in theory, however many struggled to have enough water or enough, if any, soap. "If I find the soap I can use the soap. If I find the water I can use the water" (Guerry 2013)	Acceptability/ reliability	"Sometimes you want to take a bath three times and your mother quarrel's you that you are misusing her soap." (Mason 2013)
Environment for washing materials		Cleanliness<br>Privacy<br>Safety	"Where will we wash, where will we put them for drying. You stay in a big house. You must be washing in one corner of the house. Here sometimes a man is passing, sometimes a young boy is passing, then how can we wash." (Garg 2001)<br><br>"If we wash at day time, there would be people moving around… people will look at us and will say that girl has no brains… we need a place where if we wash the cloth no one can see." (Caruso 2017)
**Drying materials**			
Location materials dried<br><br>Materials completely dry before use	"I wash the cloths at night inside my hutment when my children are asleep and spread them over my hutment roof. By morning they've become dry" (Garg 2001)<br><br>"Yes, we dry the cloth under the sunlight. But most of the time we place it under some clothes. When we do it directly then it is in a "kolte" (slanted) place - so no one can see it." (WaterAid 2009)<br><br>"Us girls in the boarding [school], we wash our menstrual cloth at night and dry them under our mattresses." (Lahme 2016)	Acceptability	"The cloths trap the rust from the iron wire and when we use it the rust enters our body and causes infections" (Morrison 2016)<br><br>"if you put them under your bed to dry, there are bacteria and it is not hygienic – and there is no iron to use on them – so it's a problem" (Sommer 2009)<br><br>Washing menstrual cloth during the rains was challenging, but drying was harder. Cloths could blow off lines, becoming dirty again or would not fully dry and be worn while damp, potentially leading to chaffed skin. (Caruso 2017)
Environment for drying materials		Privacy<br>Safety	Girls feared the embarrassment related to other people seeing their cloths drying outside. (Guerry 2013)<br><br>"In the village we were able to wash and hang the rags anywhere without shame, in the camp that is very difficult." (Parker 2014)
Materials covered while drying	laundering practices included hiding damp menstrual cloths underneath existing clothing or mattresses to dry. (Schmitt 2017)<br><br>In Fiji washed cloths are usually hung at the back of the clothes line (out of the view of neighbours), but in SI [Solomon Islands] and PNG [Papua New Guinea], women and girls spoke of hanging washed cloths out to dry under other clothes or larger pieces of material, and some even dry cloths inside the house. (IWFD 2017)	Acceptability	"(I dry) that cloth by covering up, hiding it. People will say different things…I am afraid what society will say" (Morrison 2016)
